# Optimization of a Dissolvable Lipid Nanoparticle Microneedle Formulation for mRNA Delivery Using Design of Experiments

**DOI:** 10.1021/acsami.5c05528

**Published:** 2025-07-11

**Authors:** Yayi Zhao, Qizheng Zhang, Tianli Hu, Chaiyaporn Kuwentrai, Ye Zhang, Jian-Dong Huang, Yi Kuang, Chenjie Xu

**Affiliations:** † Department of Biomedical Engineering, College of Biomedicine, 53025 City University of Hong Kong, Tat Chee Avenue, Kowloon, Hong Kong SAR, China; ‡ School of Biomedical Sciences, Li Ka Shing Faculty of Medicine, 25809 The University of Hong Kong, Pokfulam, Hong Kong SAR, China; § Department of Chemical and Biological Engineering, 121835 The Hong Kong University of Science and Technology, Clear Water Bay, Kowloon, Hong Kong SAR, China; ∥ Active Soft Matter Group, 641635 Songshan Lake Materials Laboratory, Dongguan, Guangdong 523808, China; ■ Institute of Digital Medicine, 53025 City University of Hong Kong, Tat Chee Avenue, Kowloon, Hong Kong SAR, China

**Keywords:** microneedle, lipid nanoparticle, Design of Experiments, mRNA, transdermal drug delivery

## Abstract

Microneedles (MNs) are an emerging strategy to realize the transdermal delivery of lipid nanoparticles (LNPs) in a minimally invasive manner. Via development, the LNP’s physicochemical properties, such as size and charge, and the MN’s composition and fabrication procedure, must be optimized. Currently, the optimization is done through trial and error, which is heavily influenced by personal experience and preference of researchers. This study utilizes Design of Experiments (DoE) for optimizing parameters in LNP-MN fabrication to gain independence from personal experience and preference. As a proof of concept, we develop an LNP-MN device to deliver mRNA encoding green fluorescent protein (GFP). Flow cytometric analysis reveals that freshly prepared LNP-MNs achieve a transfection efficiency of 43.3% in mesenchymal stem cells, compared with 8.51% for the lipofectamine control. The LNP-MN group also provides more homogeneous transfection (99.4% GFP positive cells), while the number is only 31.0% in the lipofectamine group. mRNA-LNP-MN maintains a transfection efficiency of 22.7% after 42 days of storage at room temperature. Finally, luciferase mRNA is successfully transfected into mice by the mRNA-LNP-MN delivery system.

## Graphical abstract

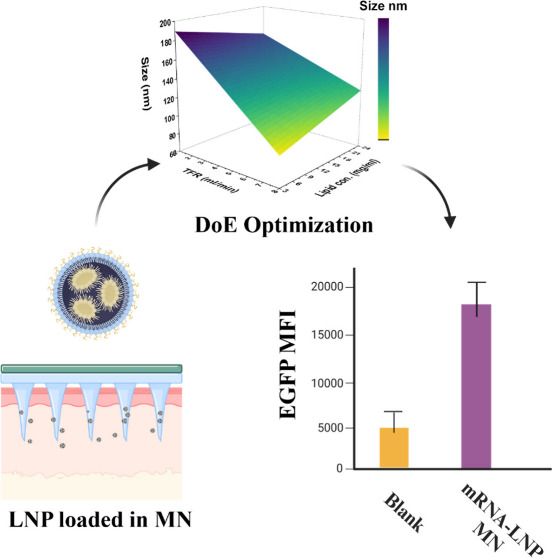

## 1 Introduction

Lipid nanoparticles (LNPs), including liposomes, solid lipid nanoparticles, nanostructured lipid carriers, and polymer–lipid hybrid nanoparticles, are ideal carriers for delivering various therapeutic agents.^1^ Initially inspired by the lipid-bilayer structure of liposomes, LNPs have evolved into more complex architectures with broad physicochemical characteristics. These advancements have significantly enhanced drug solubility, stability, and bioavailability during storage and administration while reducing toxicity and side effects *in vivo*. The approval and global application of LNP-based drugs, such as Doxil, Patisiran, and COVID-19 mRNA vaccines, have further validated their safety and efficacy.^2^ As highly efficient nonviral carriers, LNPs are considered ideal for delivering mRNA and other nucleic acids due to their excellent biocompatibility, high delivery efficiency, and structural stability.^2,3^ Despite these success, LNPs face challenges such as nonspecific distribution to off-target organs, and difficulty to cross biological barriers (skin and cell membranes).^4^ Furthermore, the conventional administration methods (i.e., intravenous or intramuscular injection) often cause patient discomfort and anxiety.^5,6,7^

Microneedles (MNs) are an emerging platform for transdermal delivery of LNPs.^4,8^ LNPs can either be embedded in the dissolvable MN matrix or coated on the surface of nondissolvable MNs. Once the MN tips penetrate the stratum corneum and reach the epidermis or dermis, LNPs are released and diffuse into subcutaneous tissues and capillaries. This strategy can help solve the challenges LNP formulations are facing such as the off-target distribution and anxiety in the adminstration. For instance, van der Straeten et al. delivered mRNA-LNP COVID-19 vaccines using polyvinylpyrrolidone (PVP) MNs,^9^ while Qu et al. utilized hyaluronic acid (HA) MNs to deliver dexamethasone-loaded cationic liposomes for psoriasis therapy.^10^ Moreover, transportation of LNPs relies on cold-chain logistics.^9^ The integration of LNPs into MNs not only improves the stability but also eliminates the need for cold-chain transportation. By loading LNPs on MNs, free water is removed, inhibiting lipid-bilayer hydrolysis and particle aggregation to preserve RNA integrity and nanoparticle’s structure.^9,11,12,13^

However, to develop an efficient LNP-MN delivery system, we must optimize the physicochemical properties of LNPs (e.g., particle size and charge) to regulate nano–bio interactions and select appropriate MN materials and fabrication techniques to ensure sufficient mechanical properties. The current optimization process is based on trial and error, heavily influenced by researchers’ personal experience and preference. As a systematic and efficient approach, Design of Experiments (DoE) allows researchers to study the relationships between multiple input and output variables, thereby developing reproducible and reliable experimental protocols. In LNP development, DoE could allows the systematic optimization of key quality attributes such as particle size, encapsulation efficiency, and ζ potential.^5,6^ It also enables precise optimization of geometric parameters (e.g., tip radius and height) and mechanical properties of MNs, ensuring effective skin penetration and structural integrity.^14,15^ For example, Safford et al. used DoE to create a library of C12-494 LNPs with varying excipient molar ratios, identifying optimal formulations for enhanced mRNA delivery to the placenta,^16^ while Held et al. optimized the fabrication process of silicon MNs using a DoE approach.^17^

This study explores the use of DoE to optimize the formulations of LNPs and MNs in the development of the LNP-MN delivery system. LNPs are synthesized using a microfluidic device, where we tune the total lipid concentration and total flow rate (TFR) to optimize the size and polydispersity index (PDI). In the MN fabrication, we investigate Young’s modulus and LNP size post the release from MNs by tuning the preparation concentration of PVP and drying temperature. Two-dimensional (2D) and three-dimensional (3D) cell models are then used to examine the transfection capabilities of free LNPs and LNP-MNs. Finally, LNP-MNs are applied on the mouse model for the transdermal delivery of luciferase mRNA. We envision this DoE-assisted approach would facilitate the development of the LNP-MN system in a systematic and objective way, preventing interference from personal experience and preference.

## 2 Results and Discussion

### 2.1 Optimization of LNPs Using the DoE Approach

LNPs, composed of four lipid components, were synthesized using a Y-shaped microfluidic device (Figure S1).^18^ We selected four lipid components to synthesize LNPs. The components included cationic lipids (2,3-dioleoyloxy-propyl)-trimethylammonium chloride (DOTAP), 1,2-dipalmitoyl-*sn*-glycero-3-phosphocholine (DPPC), 1,2-distearoyl-*sn*-glycero-3-phosphoethanolamine-*N*-[methoxy­(polyethylene glycol)-2000] (DSPE-PEG 2000), and cholesterol.^19^ Instead of commonly used ionizable lipids, the cationic DOTAP was chosen here for facilitating cellular uptake of LNPs *in vitro* and *in vivo* after the localized delivery.^20,21^ LNPs composed of ionizable lipids are more suitable for systemic delivery as they remain neutral at physiological pH.^22,23^ Fluorescein isothiocyanate-dextran (FITC-dextran, MW = 250 000 Da) was taken as the model drug in the optimization.^24^ The LNP size was between 125 and 190.4 nm (PDI ranging from 0.073 to 0.45) by tuning the total lipid concentration and the total flow rate (TFR) between the aqueous phase and organic phase (a 2^2^ full factorial design (Table S1)). The total lipid concentration directly influences the particle size and drug encapsulation efficiency of LNPs, while the TFR determines the uniformity and stability of the particles by affecting the mixing efficiency, such as the Reynolds number and shear forces.^16,25^ During the experiments, the flow rate ratio (FRR) was fixed at 3 (a common value in the literature^26^) while the DOTAP:DPPC:DSPE-PEG 2000:cholesterol molar ratio was fixed at 30:18:2:50. The LNP size and PDI are the output variables (also known as responses), while the lipid concentration and TFR are input variables (also knoown as factors). The correlation between the factors and the responses can be visually observed in the 3D plot of DoE (Figure 1).^25,27^

**Figure 1:**
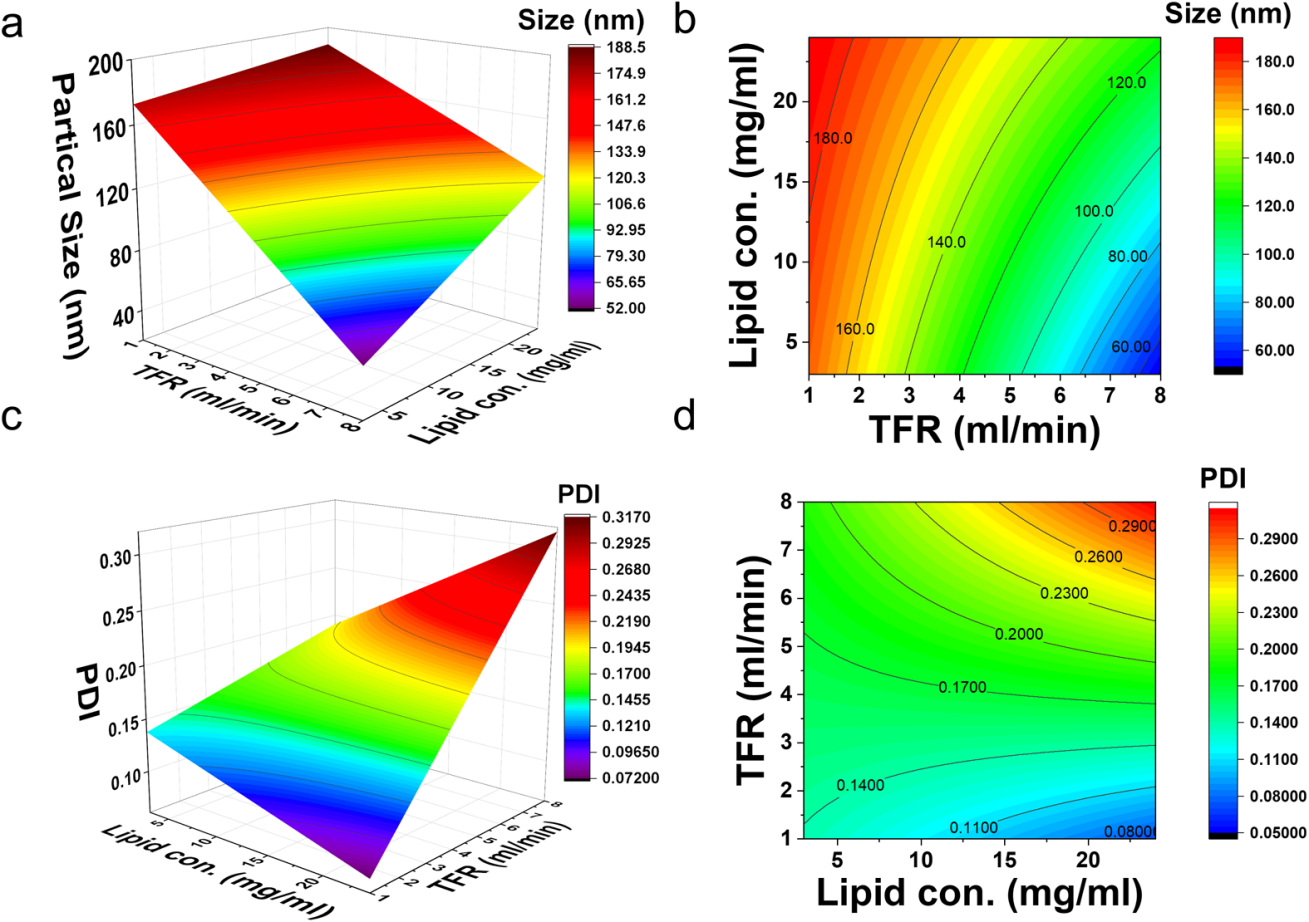
Optimization of LNP synthesis using the DoE approach. (a) Response surface plot and (b) 2D contour plot for the optimization of LNP size vs TFR and total lipid concentration. (c) Response surface plot and (d) 2D contour plot for the optimization of LNP PDI vs TFR and total lipid concentration.

The ideal size of LNPs is 120 ± 30 nm with PDI < 0.3 so that LNPs could be internalized by the cells efficiently.^28^ Figure 1a is the response surface plot of DoE for the optimization of LNP sizes with TFR versus lipid concentration (one-way analysis of variance (ANOVA); *p* < 0.0007). The predicted *R* ^2^ of 0.8891 reasonably agrees with the adjusted *R* ^2^ of 0.9668. *R* ^2^ measures the goodness of fit of a regression model to the observed data. It is between 0 and 1, where a higher *R* ^2^ value indicates a better fit of the model to the data. The LNP size decreased when the TFR increased regardless of the total lipid concentration (Figure 1a). However, the variation of the LNP size due to the changes in the TFR is more significant when the total lipid concentration is low. From the contour lines (Figure 1b), we could see that the LNP size was around 120 ± 30 nm when the TFR and total lipid concentration were in the ranges of 4–8 mL/min and 3–24 mg/mL, respectively.

Besides the LNP size, we also examined the PDI under different synthetic conditions (*p* < 0.0001). When the PDI of LNPs is less than 0.3, the size distribution of LNPs can be considered to be uniform. Regardless of the lipid concentration, the PDI increased with the TFR (Figure 1c). The variation of PDI due to the changes in TFR is more significant when the total lipid concentration is higher. However, when examining the correlation between the lipid concentration and PDI, two distinct stages can be observed. At lower flow rates (TFR < 4 mL/min), the higher the lipid concentration the smaller the PDI (Figure 1c).^29^ When the TFR is between 4 and 8 mL/min, the higher the lipid concentration the larger the PDI.^30,31^ From the contour plot (Figure 1d), we could see that the PDI remained below 0.3 as long as the TFR and lipid concentration were not simultaneously distributed in the ranges of 5–8 mL/min and 10.5–24 mg/mL, respectively. To facilitate the control of the production process, we chose a TFR between 4 and 6 mL/min and a total lipid concentration between 3 and 14 mg/mL.

To summarize, ideal LNPs with a size of 120 ± 30 nm and a narrow particle size distribution (PDI < 0.3) can be obtained when the TFR is between 4 and 6 mL/min and the lipid concentration is in the range of 3–14 mg/mL.

### 2.2 Synthesis and Characterization of FITC-Dextran-LNPs or mRNA-LNPs

When the TFR is between 4 and 6 mL/min and the total lipid concentration is between 3 and 14 mg/mL, both the LNP particle size and PDI will meet the requirements, and any value within the range can be chosen. With a total lipid concentration of 6.5 mg/mL and a TFR of 4.8 mL/min (applicable to the accuracy of the instrument), we prepared FITC-dextran-LNPs and mRNA-LNPs (EGFP-L-Cap1 AG (N1ψ), Shenzhen Dakewei Biotechnology Co.). The hydrodynamic diameters of both LNPs were around 142.47 ± 8.39 and 144.4 ± 8.03 nm (PDI = 0.219 and 0.195) with ζ potentials of 14.33 ± 0.73 and 13.9 ± 3.80 mV, respectively (Figure 2a,d). The PDI of LNPs was measured before dialysis to obtain the intrinsic heterogeneity generated during microfluidic mixing. The ζ potential in PBS was evaluated after dialysis to reflect the final physiologically relevant properties of the LNPs. Under transmission electron microscopy (TEM) (Figure 2b,c), both LNPs showed relatively homogeneous shapes with an external bilayer and an electron-dense internal amorphous structure. We also quantified the drug loading in LNPs. There was 0.3775 mg of FITC-dextran in 1 mg of FITC-dextran-LNPs and 0.02374 mg of mRNA in 1 mg of mRNA-LNPs. FITC-dextran-LNPs looked slightly denser under TEM than mRNA-LNPs because there was more FITC-dextran in LNPs.

**Figure 2:**
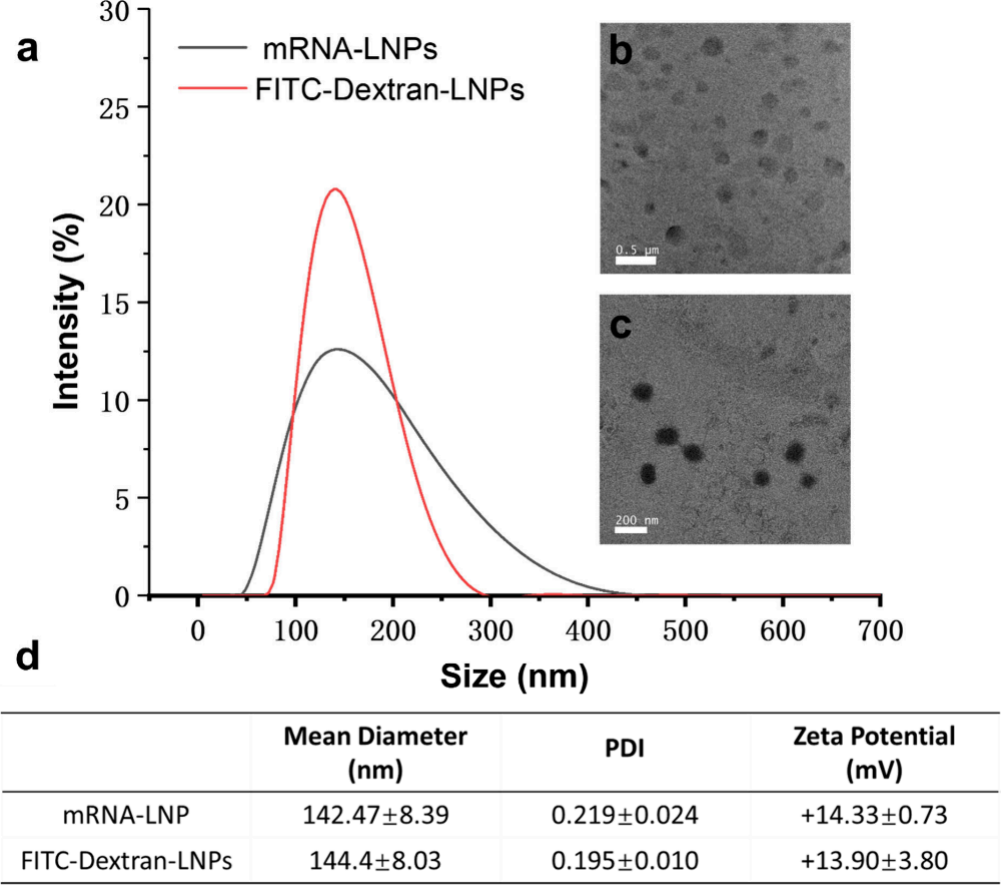
Characterization of FITC-dextran-LNPs or mRNA-LNPs. (a) Hydrodynamic diameters after dialysis. TEM images of (b) mRNA-LNPs (scale bar of 0.5 μm) and (c) FITC-dextran-LNPs (scale bar of 200 nm). (d) Quantification of LNPs’ hydrodynamic diameters and ζ potentials after dialysis.

### 2.3 Cell Transfection by LNPs

We selected mesenchymal stem cells (MSCs) and HaCaT cells (human keratinocyte cell line) as model cells to evaluate the transfection efficiency of the LNP and LNP-MNs. MSCs are multipotent cells with immune regulatory capabilities and can differentiate into distinct cell lineages.^32^ They have been used for skin repair and for improving wound healing. The successful transfection of MSCs using LNPs and LNP-MNs would provide methods for MSC engineering both in vitro and *in vivo*. HaCaT cells represent keratinocytes in the skin’s epidermal layer.^33^ Its transfection reflects the actual performance of LNPs and LNP-MNs in transdermal drug delivery.^34^

MSCs were transfected with FITC-dextran-LNPs, mRNA-LNPs, FITC-dextran, or mRNA alone as a negative control, or commercial lipofectamine-loaded drugs as a positive control. We first examined the results of EGFP mRNA transfection through fluorescence imaging (Figure 3a–c). Lipofectamine- and LNP-transfected cells became fluorescent, although the lipofectamine group showed stronger average fluorescence intensity per fluorescent cell (Figure 3d). In contrast, negative control cells showed no detectable EGFP fluorescence. These cells were further examined through flow cytometry analysis (Figure 3e–g). Interestingly, in the mRNA-LNP group, there were 99.4% EGFP positive cells, while the number was only 31.0% in the lipofectamine group (Figure 3h). Flow cytometry allows for single-cell fluorescence intensity measurements across large cell populations, providing absolute quantitative data while avoiding biases associated with image-based analysis. Therefore, LNPs provided a more homogeneous transfection while lipofectamine delivered more molecules into a smaller portion of cells. Therefore, the positive lipofectamine group (Figure 3f) contained cells with a wider range of fluorescence intensity than the LNP group (Figure 3g). To directly assess the cellular uptake and endosomal/lysosomal escape of mRNA-LNPs in MSCs, we employed FITC-dextran-labeled LNPs as a surrogate and performed dual staining with Hoechst and LysoTracker Deep Red (Thermo Fisher) 1, 3, and 12 h after treatment, followed by confocal microscopy (green, FITC-dextran-LNP; red, LysoTracker; blue, Hoechst). At 1 h, the green and red fluorescence signals overlapped extensively, appearing as yellow puncta, indicating that the majority of LNPs remained sequestered within endosomal/lysosomal compartments. By 3 h, discrete green puncta began to dissociate from the red-labeled organelles, signifying the initiation of endosomal escape. At 12 h, a substantial proportion of green fluorescence was observed throughout the cytosol, with markedly reduced colocalization with the red signal, confirming that most LNPs had successfully escaped into the cytosol (Figure S10). This time-dependent redistribution of FITC-dextran-LNPs corroborates their endosomal escape capability in MSCs.

**Figure 3:**
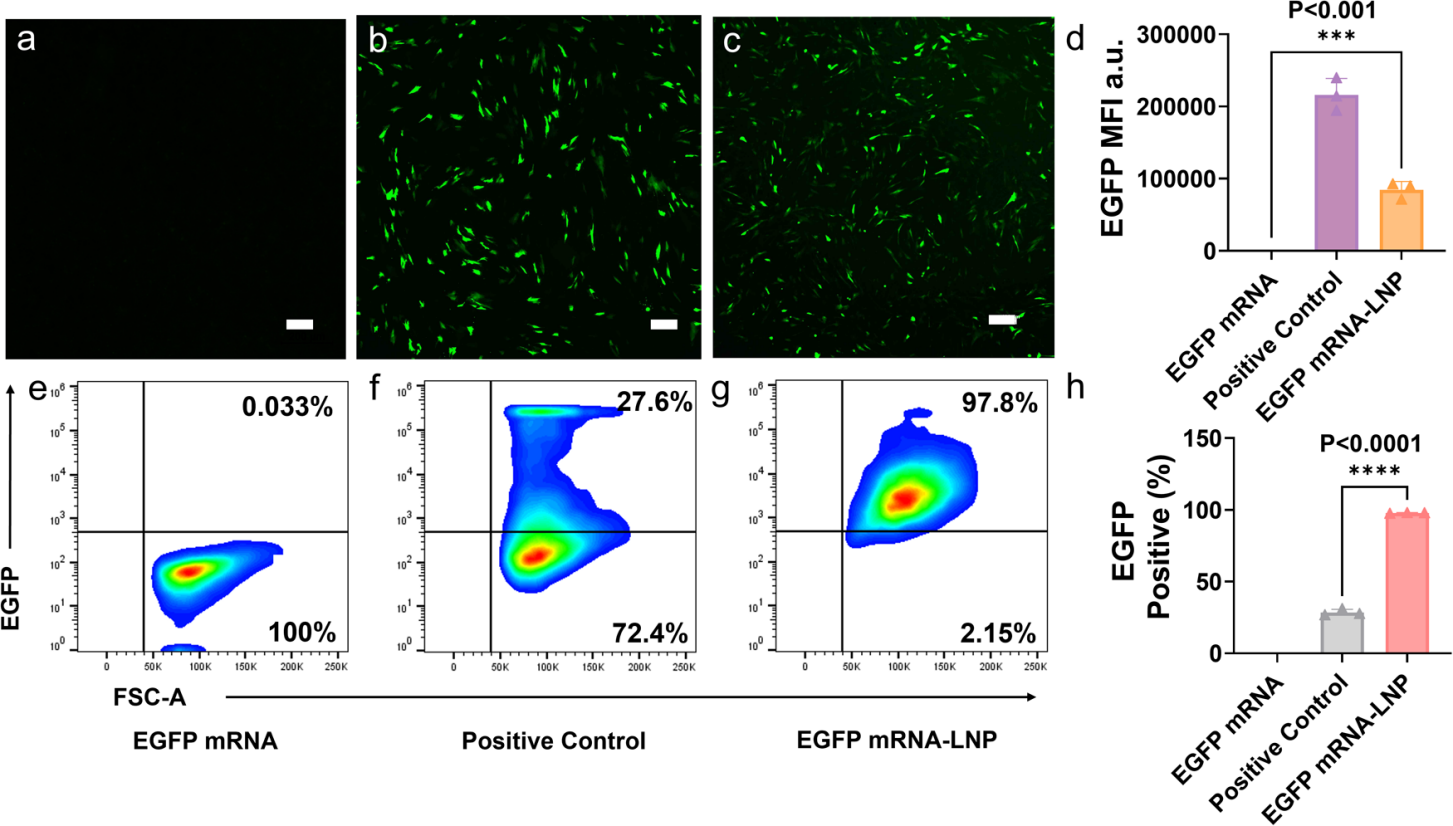
MSCs transfection by EGFP-mRNA-LNPs. Fluorescence imaging of MSCs transfected with (a) EGFP mRNA alone, (b) EGFP mRNA-lipofectamine, and (c) EGFP mRNA-LNPs (scale bars of 250 μm). (d) Quantification of the average fluorescence per fluorescent cell in the fluorescence image. Mean fluorescence intensity (MFI) is a unit used to quantify the fluorescence intensity of EGFP expression in cells observed via fluorescence microscopy. Flow cytometry analysis of MSCs transfected with (e) EGFP mRNA alone, (f) EGFP mRNA-lipofectamine, and (g) EGFP mRNA-LNPs. (h) Quantification of the fluorescence intensity of cells in flow cytometry. Values are expressed as the mean ± SD. Error bars indicate SD values from three independent experiments. Statistical differences are expressed as follows: *p < 0.05,**p < 0.01,***p < 0.001, and ***p < 0.001.

When FITC-dextran was delivered, lipofectamine and LNPs showed totally different results (Figure S2). In both fluorescence imaging (Figure S2a–d) and flow cytometry analysis (Figure S2e–g), lipofectamine did not deliver FITC-dextran into the cells. In contrast, LNPs successfully transfected cells with FITC-dextran. Similar to the results for mRNA transfection, the transfection was also homogeneous (Figure S2g). The failure of FITC-dextran transfection using lipofectamine can be explained by the ζ potential of the FITC-dextran-lipofectamine complex. In mRNA transfection, the EGFP-mRNA-lipofectamine showed a ζ potential of 6.310 mV in DMEM, which would allow the complexation between the cell membrane and EGFP-mRNA-lipofectamine conjugate. However, FITC-dextran-lipofectamine conjugates showed a ζ potential of −1.820 mV in DMEM, which would not facilitate the complexation. We also successfully transfected skin fibroblasts and HEK 293 cells using FITC-dextran-LNPs (Figure S3).^35^ This comparison guides the optimization of future LNP designs, particularly in evaluating the efficacy of specific components (e.g., cationic lipids or PEG modifications) for nucleic acid delivery, and offers direction for developing specialized LNP platforms tailored for different small-molecule drugs.

In the case of HaCaT cells, both lipofectamine and LNP groups are fluorescent. However, the transfection of lipofectamine was much less efficient than that of LNPs (Figure 4d). The flow cytometry analysis was consistent with the imaging results (Figure 4e–g). Minimal fluorescence signals were detected in both the negative control group and the lipofectamine group (Figure 4e,f). In contrast, 51.37% of cells in the mRNA-LNP group were EGFP positive, while only 0.33% of cells in the lipofectamine group showed EGFP expression (Figure 4f–h).

**Figure 4:**
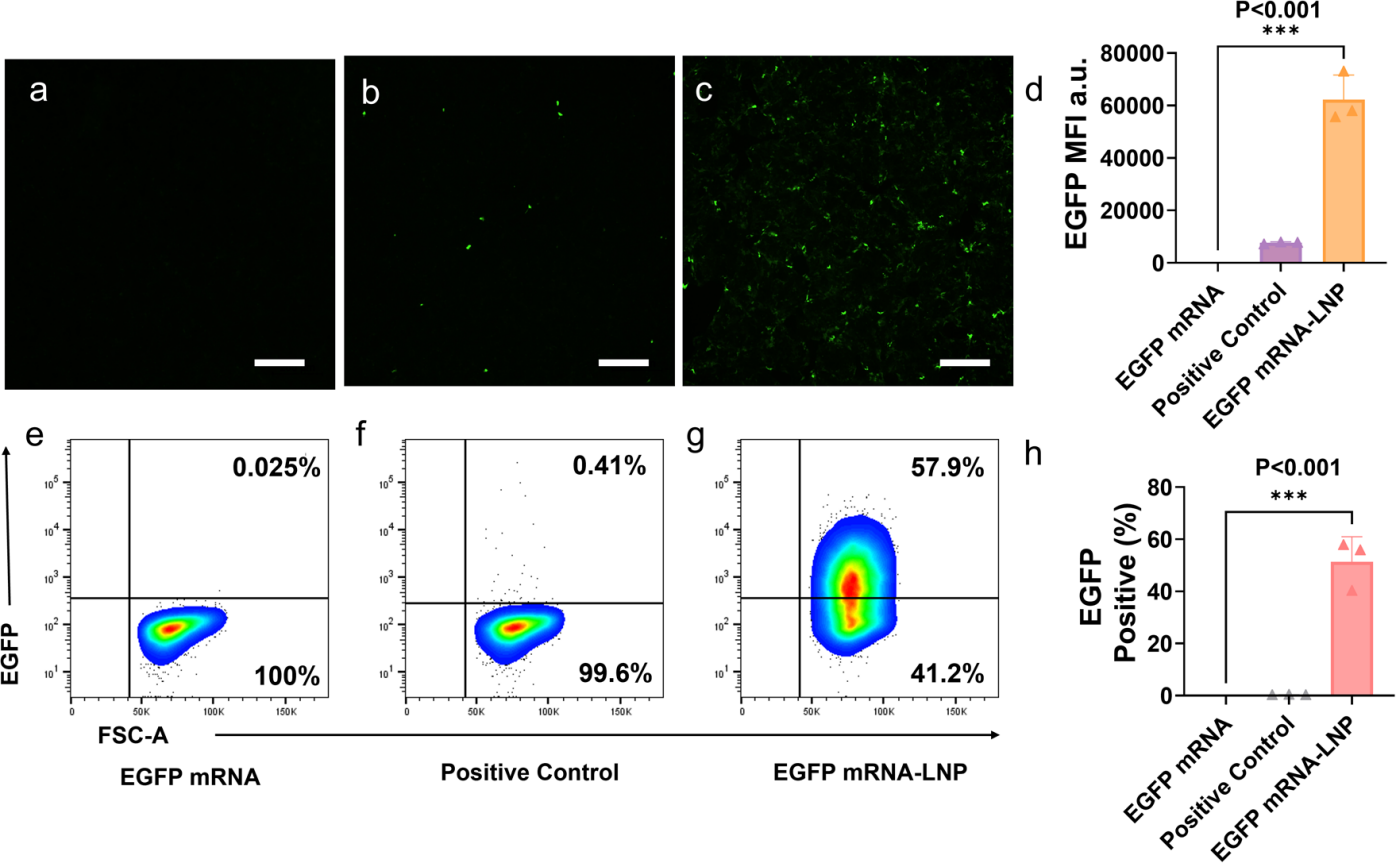
HaCaT transfection by EGFP-mRNA-LNPs. Fluorescence imaging of HaCaT cells transfected with (a) EGFP mRNA alone, (b) EGFP mRNA-lipofectamine, and (c) EGFP mRNA-LNPs (scale bars of 200 μm). (d) Quantification of the average fluorescence per fluorescent cell in the fluorescence image. MFI (mean fluorescence intensity) is a unit used to quantify the fluorescence intensity of EGFP expression in cells observed via fluorescence microscopy. Flow cytometry analysis of HaCaT transfected with (e) EGFP mRNA alone, (f) EGFP mRNA-lipofectamine, and (g) EGFP mRNA-LNPs. (h) Quantification of the fluorescence intensity of cells in flow cytometry. Values are expressed as the mean ± SD. Error bars indicate SD values from three independent experiments. Statistical differences are expressed as follows:*p < 0.05, **p < 0.01, ***p < 0.001 , and ****p < 0.0001.

In this section, EGFP mRNA-LNP was used to evaluate the efficiency of LNP in delivering nucleic acids (mRNA), involving multiple steps such as cellular uptake, endosomal escape, and the translation of mRNA into protein.^3,36^ FITC-dextran-LNP served as a model molecule to assess the efficiency and distribution of LNPs in delivering non-nucleic acid molecules (e.g., macromolecular carbohydrates), requiring only unpacking and cytoplasmic release. These provide a thorough understanding of LNP’s capability in delivering nucleic acids versus non-nucleic acid molecules.^37^

### 2.4 Optimization of Dissolvable LNP-MN Formulations Using the DoE Approach

We chose PVP to fabricate the MN patch because of its quick dissolution in skin and capability to preserve mRNA stability.^38^ Its hygroscopic nature regulates the microenvironment’s humidity, preventing degradation of sensitive molecules like mRNA due to excessive moisture.^39^ Its film-forming properties can create a protective barrier around the drugs, effectively shielding it from oxygen, temperature fluctuations, and enzymatic degradation. Furthermore, its inertness and biocompatibility ensure no chemical reactions with the drug molecules, thereby preserving their activity to a maximum extent. Finally, the thermal stability of PVP prevents heat-induced degradation during the manufacturing process, making it an ideal material for ensuring exceptional drug stability.^40^

LNP-MNs were fabricated through the template molding method,^41^ where the solution containing PVP and LNPs was filled into the negative MN mold before drying (Figure S4). The key parameters here were the concentration of the polymer (i.e., PVP)^9^ and the drying temperature (input variables, also known as factors) (a 3 × 2^2^ + 1 full factorial design (Table S2)).^42^ The output variables (also known as responses) are Young’s modulus of LNP-MNs and the sizes of LNPs after the dissolution of LNP-MNs. Young’s modulus (Table S2) was derived from the force–displacement curve of the compression test (Figure S5). The slope of the displacement versus force increases rapidly at a certain point, and the force increases instantaneously at a moment that indicates that the needle has been bent.^43^ Therefore, the slope before this point is selected to estimate Young’s modulus. The total height of each MN is 700 μm; the diameter of a single tip is around 25 μm, and each patch has 100 tips, based on the following formula to calculate Young’s modulus of the tip.

$$E=\frac{\mathrm{stress}}{\mathrm{strain}}=\frac{F/A}{\Delta l/l_{0}}$$

The response surface plot of the released LNP size under different PVP concentrations (10–30%) and drying temperatures (20, 40 and 60 °C) is shown in Figure 5a. The ideal LNP-MNs should have a high Young’s modulus, and LNPs released from LNP-MNs have the same hydrodynamic diameter as before encapsulation in MNs (140 nm, Figure 2d). Across all of the drying temperatures, a lower PVP concentration is beneficial for maintaining the particle sizes (Figure 5a). This is presumably because the high viscosity of the solution at higher PVP concentration promoted LNP aggregation. From the contours (Figure 5b), it is notable that the size of dissolved LNP is less than 180 nm when the PVP concentration is between 24% and 30% and the drying temperature is between 22 and 60 °C.

**Figure 5:**
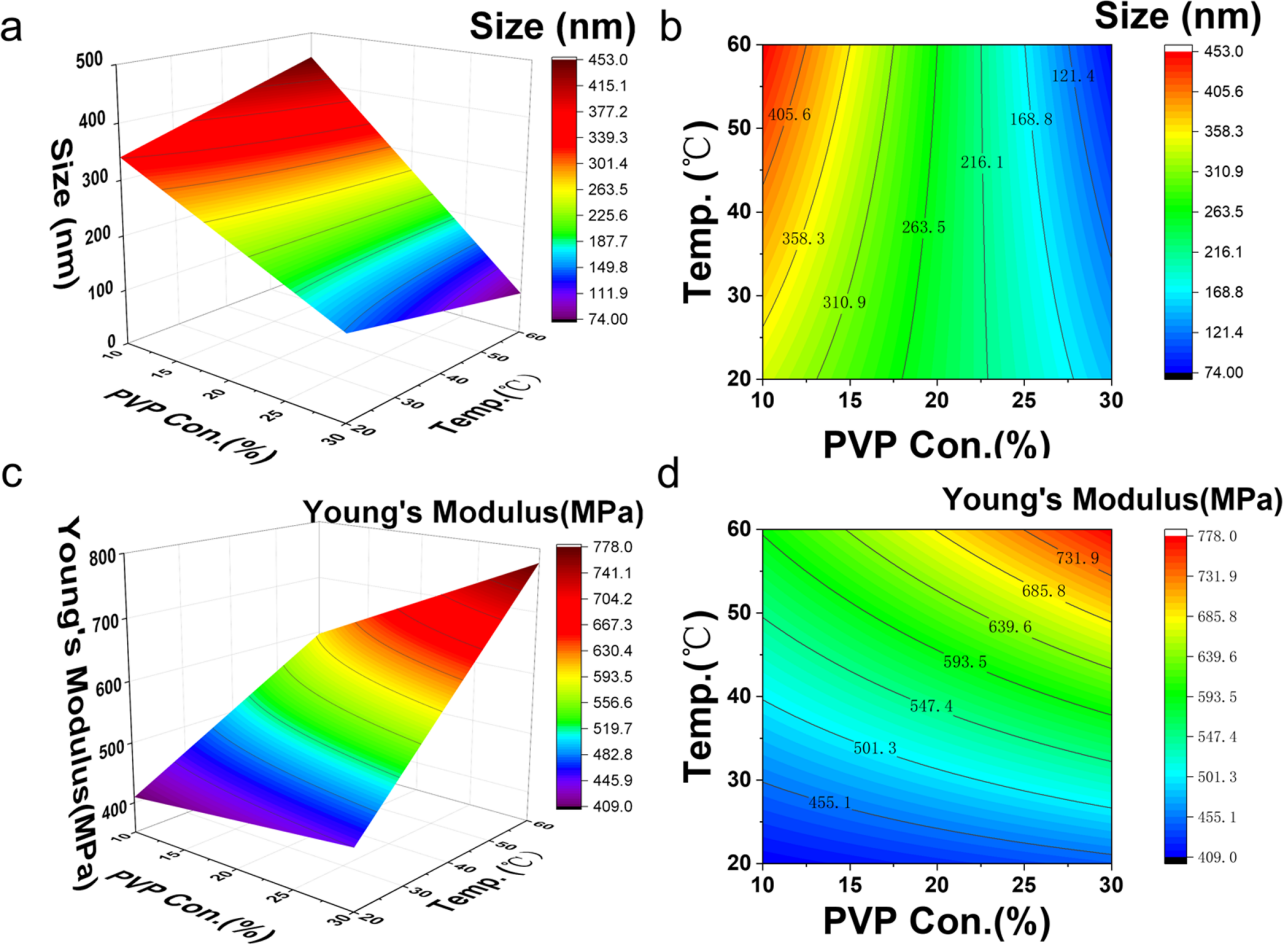
Optimization of LNP-MN formulation using DoE approaches. (a) Response surface plots of the experimental design of the PVP concentration and drying temperature on dispersibility (represented by the main particle size, the median of the range of most particle size distributions). (b) 2D contour plots of the PVP concentration and drying temperature on dispersibility. (c) Effect of PVP concentration and drying temperature on Young’s modulus of PVP needle tips. (d) 2D contour plot of the PVP concentration and drying temperature on Young’s modulus of PVP needle tips.

In addition to the dispersion of LNP-MN, we also examined the impact of different manufacturing conditions of the process on Young’s modulus of MNs. As shown in panels c and d of Figure 5, the mechanical properties are affected by the PVP concentration and drying temperature. When the PVP concentration is between 10% and 30%, Young’s modulus increases with the drying temperature. At temperatures between 20 and 60 °C, Young’s modulus increases with the PVP concentration. As mRNA is sensitive to high temperatures, we therefore chose to use LNP-MNP with a higher Young’s modulus made at low drying temperatures.

Taken together, we chose a concentration of 30% PVP and a drying temperature of 25 °C to fabricate LNP-MN. Under this condition, Young’s modulus of the needle tip is around 490 MPa and the load force per MN patch (100 tips) can be as high as 15 N (Figure S5), which is greater than the minimum average force required for normal skin penetration (5.8 N).^44^ There was no change in the LNP size through the encapsulation and release process. We evaluated the skin penetration ability of the prepared LNP-MNs on pig ear skin.^45^ As shown in Figure S6a, each MN tip produced an insertion wound. Histological analysis (Figure S6b) showed that the MN tip penetrated through the stratum corneum and reached the dermis layer.

### 2.5 Fabrication and Characterization of the Microneedle Patch (MP) Loaded with FITC-Dextran-LNPs and mRNA-LNPs

We prepared PVP microneedles loaded with mRNA-LNP and PVP microneedles with FITC-dextran-LNP according to the microneedle fabrication conditions selected above and characterized. Scanning electron microscopy (SEM) (Figure 6a–c) clearly shows that the prepared dissolvable microneedle arrays were in the shape of regular pyramids, and the height of the microneedles was about 700 μm. The surface of the needle was smooth and free of fracture, and the needle tips appeared to be sharp at high magnification of the needle tip area (Figure 6 b). Their sizes and shapes were suitable for penetrating the stratum corneum and dissolving rapidly after contact with the skin. Figure 6d presents the cumulative release profile of FITC-dextran-LNP-loaded dissolvable microneedles in PBS. The release curve follows a characteristic biphasic kinetics. During the first hour, release is rapid, with approximately 75% of the LNPs dissolved and released from the microneedles; this is followed by a slower release phase, reaching about 85% cumulative release at 4 h. Over the subsequent 20 h, the drug continues to be released, approaching complete release by 24 h. This release profile indicates that the microneedles can achieve efficient payload delivery quickly while also providing sustained release, thus combining rapid onset with prolonged dosing. We also evaluated the penetration ability of the prepared LNP-MNs in pig ear skin.^45^ Each MN tip produced an insertion wound, as shown in Figure 6f. Hematoxylin and eosin staining as well as histologic analysis showed that the MN tips penetrated the stratum corneum and reached the dermis. The resulting micropores measured approximately 310 μm in depth.

**Figure 6:**
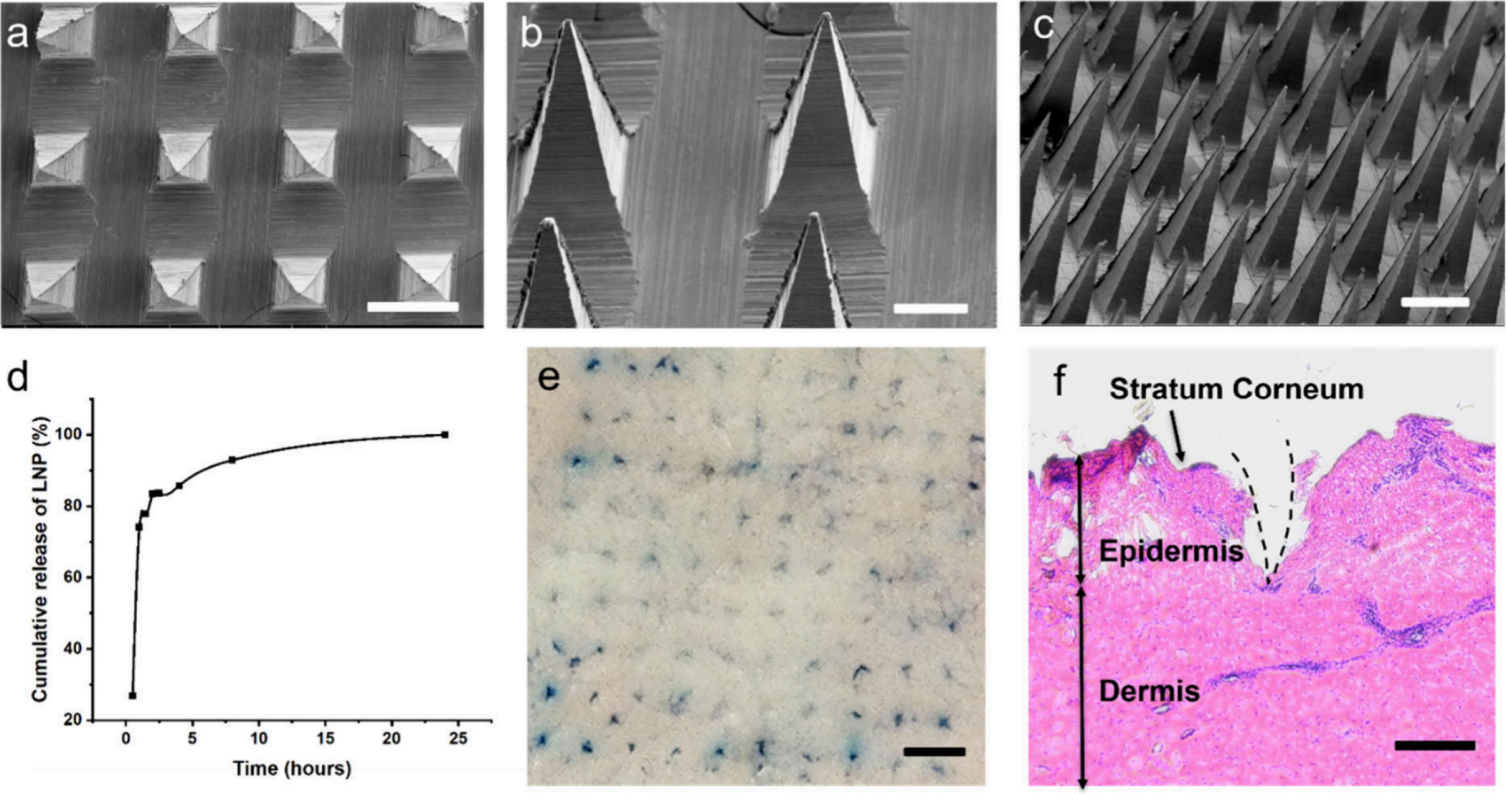
(a) Scanning electron microscopy (SEM) images of the dissolvable microneedle array. Top-down view showing the 4 × 3 needle array (scale bar of 500 μm). (b) Side view highlighting the needle height (700 μm) and smooth surfaces (scale bar of 200 μm). (c) Oblique side view of the tip (scale bar of 500 μm). (d) Cumulative release profile of LNPs from dissolvable microneedles in PBS at 37 °C. Data points represent the mean ± SD (*n* = 3). (e) Skin penetration by LNP-MNs: Trypan blue staining of MN-applied pig ear skin (scale bar of 800 μm). (f) Micrograph of hematoxylin- and eosin-stained tissue slice of pig skin following insertion of dissolvable LNP-loaded microneedles (scale bar of 200 μm).

### 2.6 Cell Transfection by LNP-MNs

These optimized LNP-MNs were examined through MSCs (Figure 7) and HaCaT (Figure 8) transfection similar to the LNPs. FITC-dextran or mRNA alone was used as the negative control. Commercial lipofectamine-loaded mRNA was used as the positive control. *In vitro* transfection experiments with EGFP-mRNA-LNP-MN were performed, as shown in Figure S6

**Figure 7:**
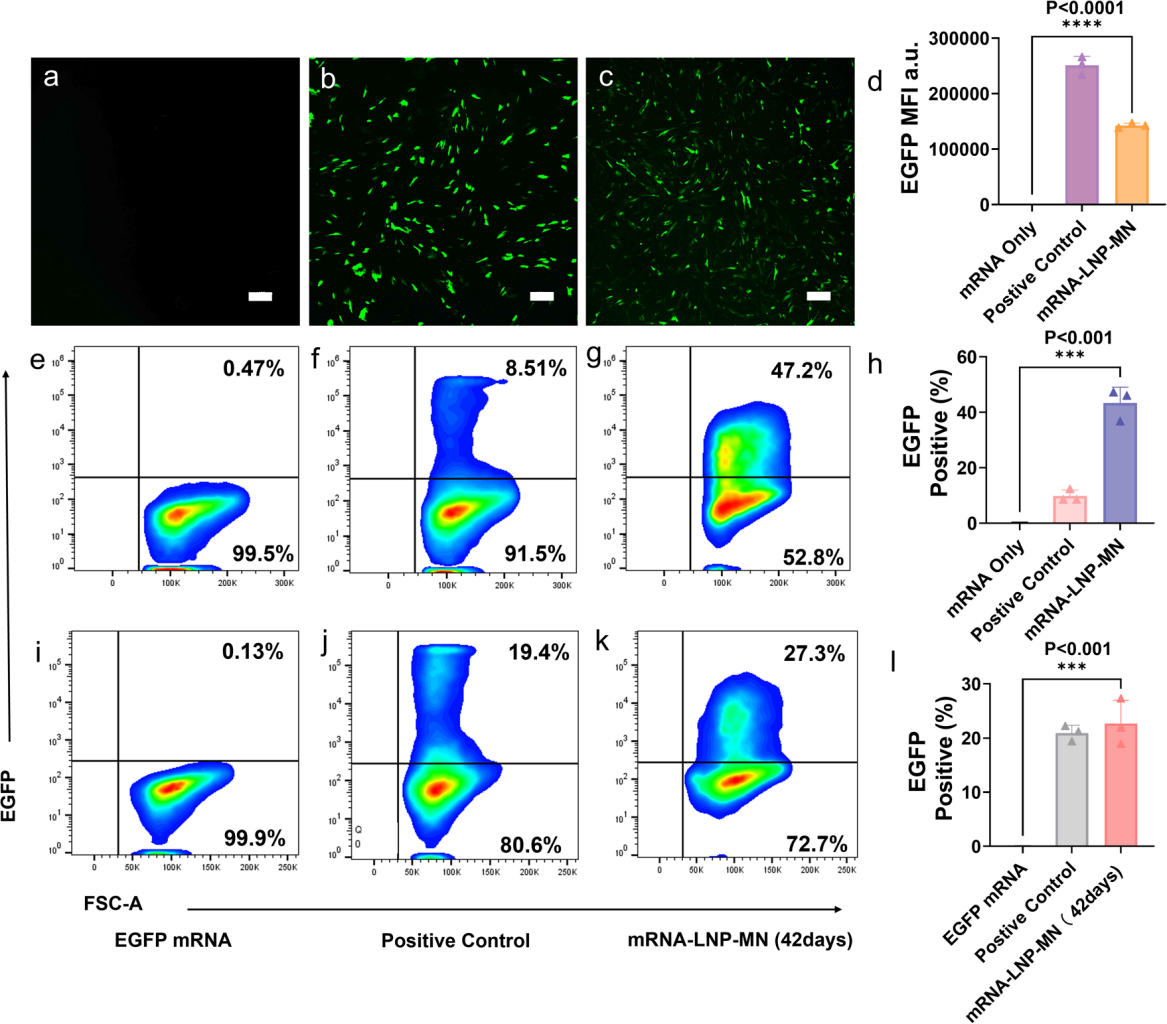
MSC transfection by EGFP mRNA-LNP-MNs. Fluorescence images of MSCs transfected with (a) EGFP mRNA alone, (b) EGFP mRNA-lipofectamine, and (c) EGFP mRNA-LNP-MNs (scale bars of 250 μm). (d) Quantification of the average fluorescence per fluorescent cell in the fluorescence image. Flow cytometry analysis of MSCs transfected with (e) EGFP mRNA alone, (f) EGFP mRNA-lipofectamine, and (g) EGFP mRNA-LNP-MNs. (h) Quantification of the fluorescence intensity of cells in flow cytometry. Values are expressed as the mean ± SD. Error bars indicate SD values from three independent experiments. Flow cytometry analysis of MSCs transfected with (i) EGFP mRNA alone, (j) EGFP mRNA-lipofectamine, and (k) EGFP mRNA-LNP-MNs stored at room temperature for 42 days. (l) Quantification of the fluorescence intensity of cells in flow cytometry. Values are expressed as the mean ± SD. Error bars indicate SD values from three independent experiments. Statistical differences are expressed as follows: *p < 0.05,**p < 0.01, ***p < 0.001andand ****p < 0.0001.

**Figure 8:**
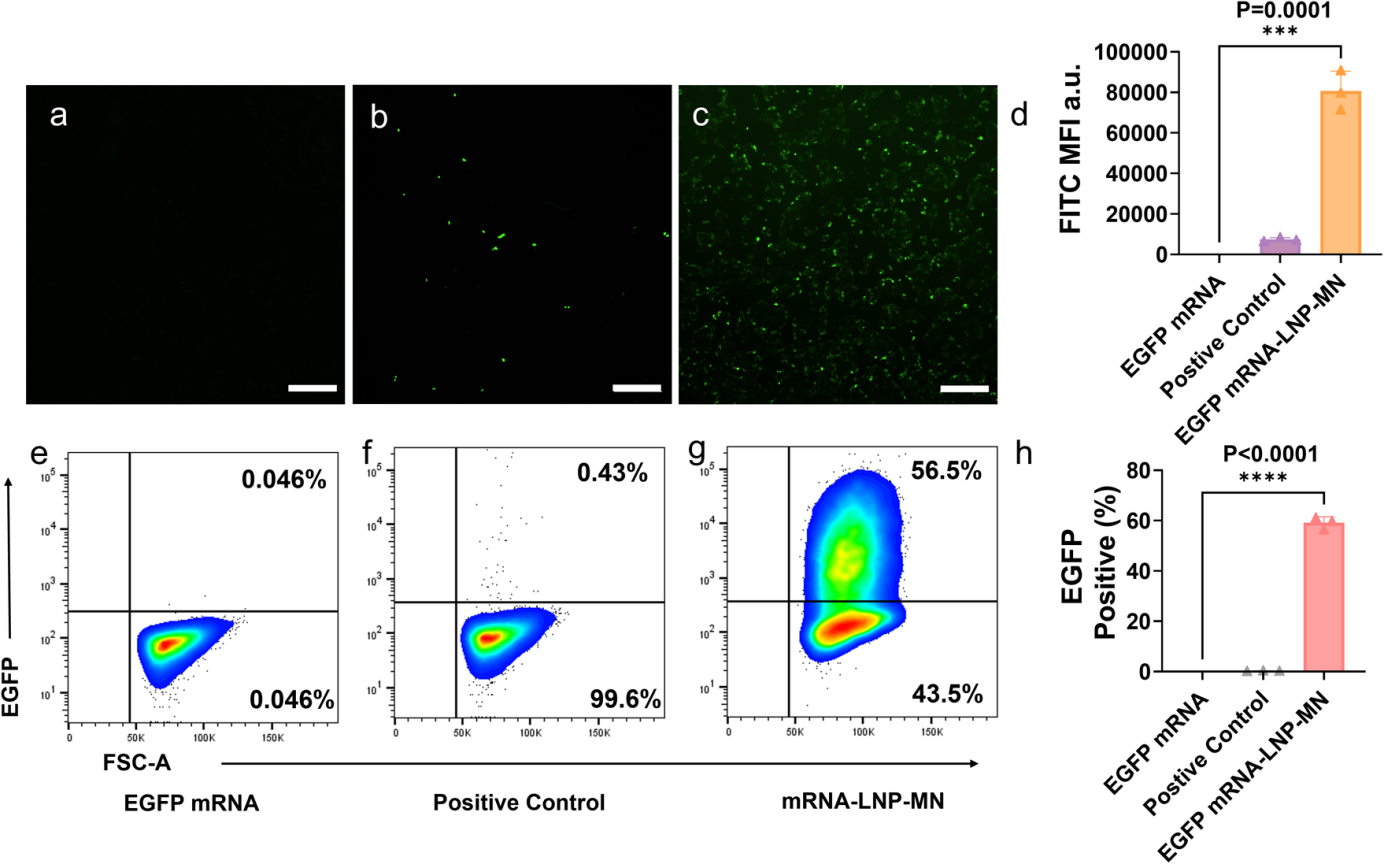
HaCaT transfection by EGFP mRNA-LNP-MNs. Fluorescence images of HaCaT cells transfected with (a) EGFP mRNA alone, (b) EGFP mRNA-lipofectamine, and (c) EGFP mRNA-LNP-MNs (scale bars of 500 μm). (d) Quantification of the average fluorescence per fluorescent cell in the fluorescence image. Flow cytometry analysis of HaCaT cells transfected with (e) EGFP mRNA alone, (f) EGFP mRNA-lipofectamine, and (g) EGFP mRNA-LNP-MNs. (h) Quantification of the fluorescence intensity of cells in flow cytometry. Values are expressed as the mean ± SD. Error bars indicate SD values from three independent experiments. Statistical differences are expressed as follows: *p < 0.05,**p < 0.01, ***p < 0.001and ****p < 0.0001.

In the case of MSCs, cells transfected with EGFP-mRNA-LNP-MNs and the positive control sample both showed clear fluorescence (Figure 7a–c). The fluorescence intensity of the cells transfected with dissolved mRNA-LNP-MNs (Figure 7d) was close to that of the cells transfected with mRNA-LNPs (Figure 3d), suggesting that mRNA-LNPs maintained their properties in the MNs. Flow cytometry analysis (Figure 7e–h) showed that mRNA-LNP-MN exhibited a transfection efficiency of 43.3%, which is substantially higher than that of the positive control group (8.51%). This suggests that LNP-MN also provided a more homogeneous transfection than lipofectamine. In addition, the manufactured mRNA-LNP-MN patches were stored for 42 days in sealed centrifuge tubes under dry air. Then they achieved a transfected efficiency of 22.7% on MSCs after 42 days of storage (Figure 7i–l).

Still on MSCs, LNP-MNs successfully delivered FITC-dextran into the cells, proven through both fluorescence imaging (Figure S7a–d) and flow cytometry (Figure S7e–g). There was an extremely high transfection efficiency of more than 95% with FITC-dextran-LNP-MN, which proved that LNP encapsulated in PVP soluble MNs could be released well and subsequently internalized by cells. We also successfully transfected fibroblasts using FITC-dextran-LNP-MN fabricated at a high temperature of 60 °C (Figure S8).^38^

We further examined the transfection of HaCaT cells. Optimized LNP-MNs performed similarly to LNPs (Figure 8a–c). The positive control group showed only a slight fluorescence (Figure 8b). The fluorescence intensity of cells transfected with solubilized mRNA-LNP-MNs (Figure 8d) was close to that of cells transfected with mRNA-LNPs (Figure 4d). Flow cytometry analyses (Figure 8e–h) showed that the transfection efficiency of mRNA-LNP-MNs was 59.17%, which was considerably higher than that of lipofectamine (0.31%). This indicated that mRNA-LNPs were well maintained in MNs and maintained a good transfection effect in HaCaT cells.

### 2.7 Transdermal Delivery by LNP-MNs

Besides above 2D cell models, we also constructed a 3D cell model by mixing MSCs with the xeno-free hydrogel (VitroGel MSC) (Figure 9a).^46^ The 3D hydrogel cell culture environment mimicked the physiological intradermal environment *in vivo*. MSCs in this 3D environment were transfected using LNP-MNs. We did not insert full needles, which would have distorted the thin fragile hydrogel and made it difficult to precisely insert the PVP microneedles onto the hydrogel because they would dissolve immediately upon placement in DMEM. We completely dissolved the MNs in DMEM and then added the solution to the scaffolds for coincubation, which preserved the structure of the scaffolds and reproduced the transfection of mRNA-LNP in subcutaneous cells. There were the negative control (mRNA only (Figure 9b)) and positive control (lipofectamine (Figure 9c)). Compared with the lipofectamine group (7.5%), there were many more fluorescent cells in the LNP-MN group (24.57% (Figure 9e)). However, the population is much smaller than that of LNP-MN achieved in the 2D group (43.3% (Figure 7h)). This should be due to the 3D network of the hydrogel that hindered the diffusion and migration of mRNA-LNPs after being released from MNs. Additionally, the longer and slower diffusion pathways in the 3D setting substantially reduced the local concentration of LNPs near the target cells, thereby diminishing the transfection efficiency.^47,48^

**Figure 9:**
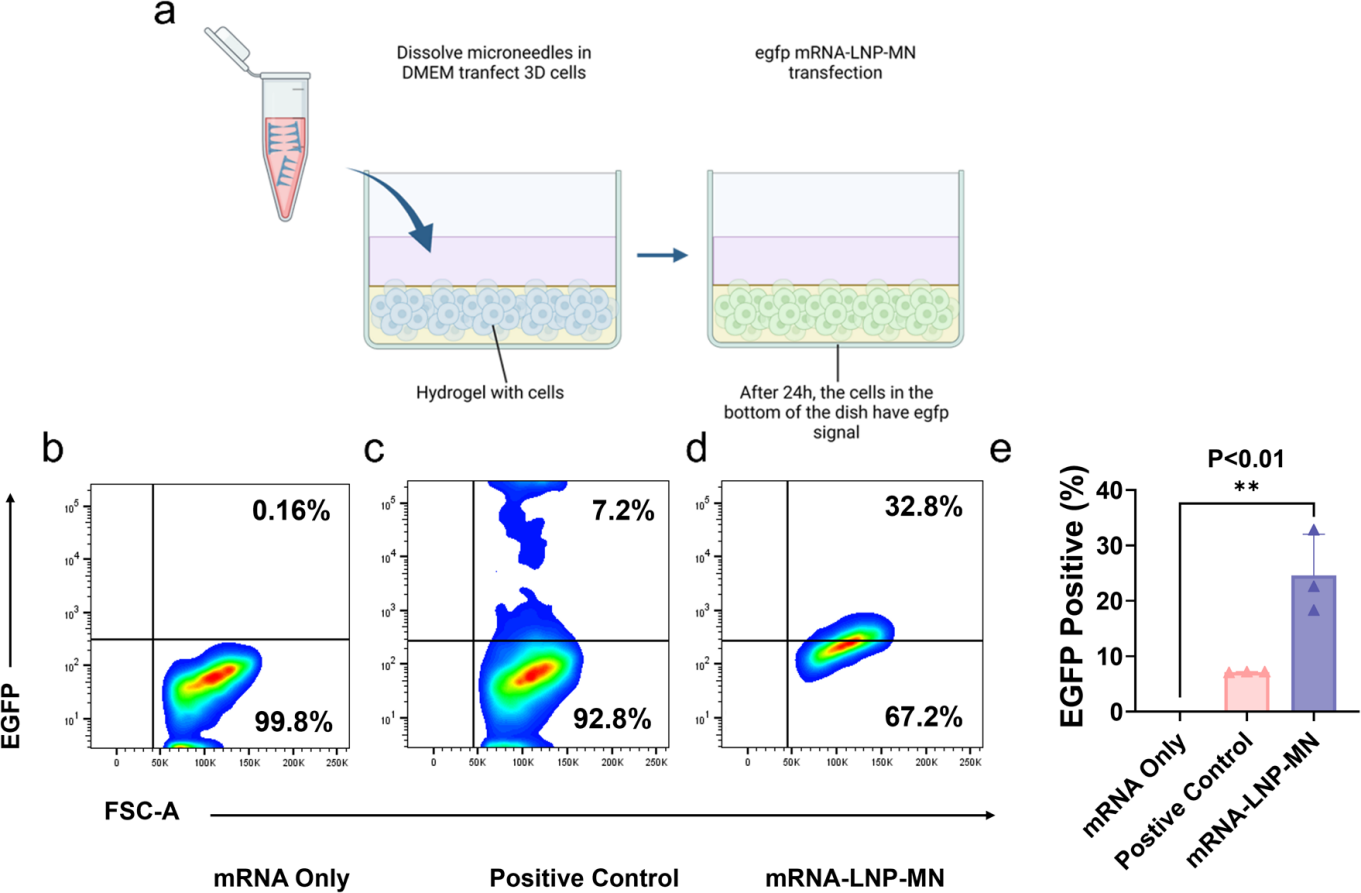
3D MSC transfection by EGFP mRNA-LNP-MNs. (a) Illustration of MSC transfected with mRNA-LNP-MNs in a 3D hydrogel. The cartoon was created in BioRender (https://BioRender.com/d93u393). Flow cytometric analysis of 3D MSCs transfected with (b) EGFP mRNA alone, (c) EGFP mRNA-lipofectamine, and (d) EGFP mRNA-LNP-MNs. (e) Quantification of the fluorescence intensity of cells in flow cytometry. Values are expressed as the mean ± SD. Error bars indicate SD values from three independent experiments. Statistical differences are expressed as follows: *p < 0.05,**p < 0.01, ***p < 0.001and ****p < 0.0001.

### 2.8 *In Vivo* Evaluation of mRNA-LNP-Loaded MNPs

We evaluated the effect of using LNP-MNs to deliver luciferase mRNA (FLUC-L-Cap1 AG (N1ψ), Shenzhen Dakewei Biotechnology Co.) on the dorsal skin of mice. After administration of mRNA-LNP-MNs, luciferase expression was monitored for 24 h using *in vivo* bioluminescence imaging (IVIS). As shown in panels a and b of Figure 10, luciferase was successfully expressed locally at the MN application site (average radiance of 4324.67 × 10^6^), in comparison to the blank group (average radiance of 2145 × 10^6^). No significant discomfort or adverse effects were observed, suggesting that mRNA-LNP-MNs were well tolerated. In the future, we will investigate the biodistribution of mRNA-LNPs after MN delivery, including the subcutaneous migration and movement of mRNA-LNPs with different particle sizes.

**Figure 10:**
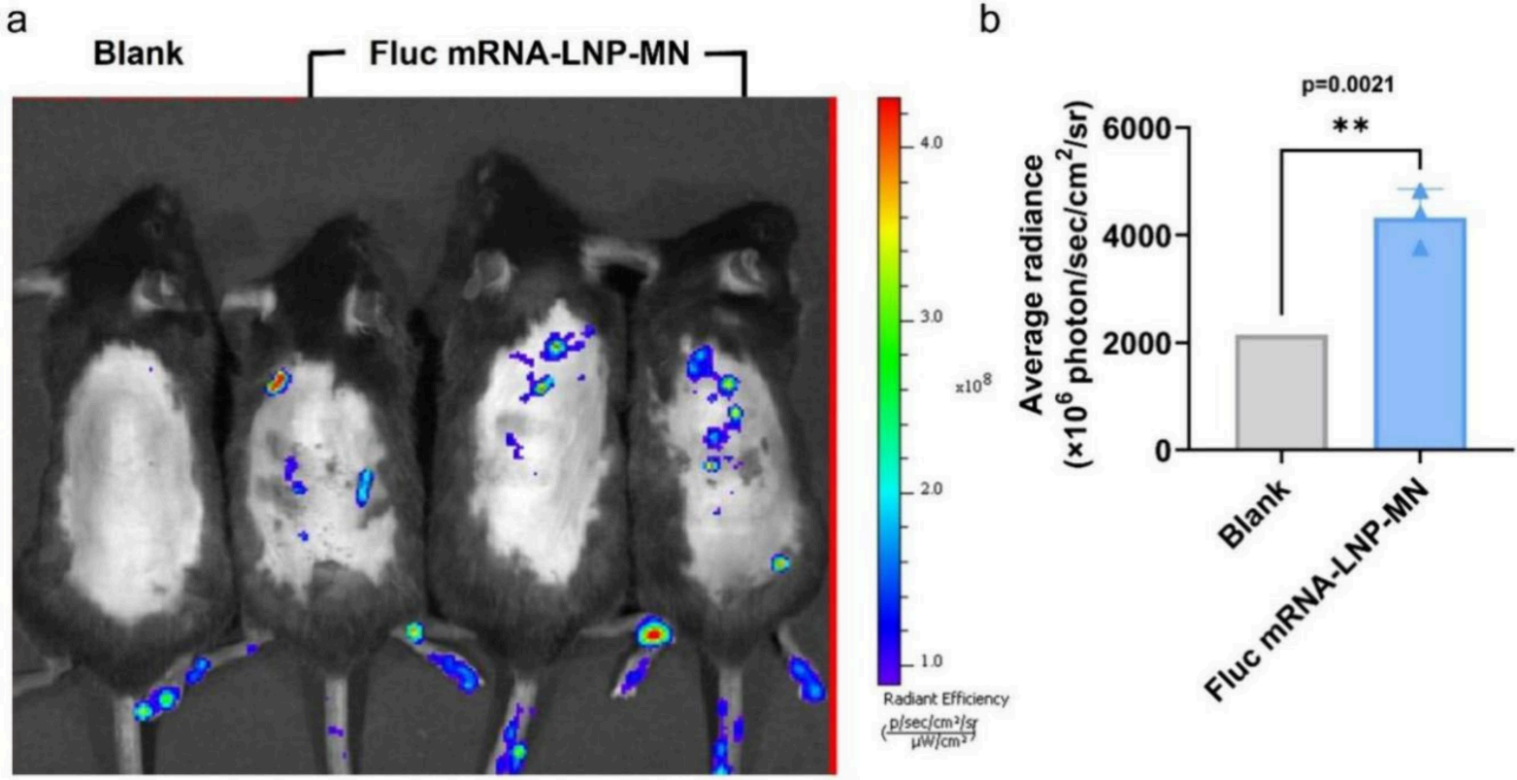
*In vivo* transfection of Fluc-mRNA using LNP-MNs. (a) Representative bioluminescence images from left to right of the blank control group and dorsal administration of Fluc mRNA-LNP-MN in mice after transdermal delivery of luciferase mRNA for 24 h. (b) Quantification of the bioluminescence signal intensity. All values are expressed as the mean of separate samples (for Fluc mRNA-LNP-MNs, *n* = 3 mice), and error bar correspond to the standard deviation (*p < 0.05; **p < 0.01; ***p < 0.001 (unpaired *t* test)).

## 3 Conclusion

This study explored the usage of DoE to optimize the formulation of LNPs and LNP-MNs for the delivery of two distinct biological molecules, nucleic acid (e.g., mRNA) and non-nucleic acid biomoleulces (e.g., FITC-dextran). DoE was used to increase the efficiency of screening, to save time, and to provide a generalized protocol.^49^ LNPs were synthesized using a microfluidic device, where we tuned the total lipid concentration and TFR to optimize the size and PDI of LNPs. In MN fabrication, we investigated Young’s modulus and LNP size after the release from MNs by tuning the preparation concentration of PVP and drying temperature. The ultimate LNPs were produced using a total lipid concentration of 6.5 mg/mL and a TFR of 4.8 mL/min, and LNP-MNP was fabricated using a PVP concentration of 30% at 25 °C. Using both 2D and 3D cell models, we showed that both free LNPs and LNP-MNs could successfully transfect MSCs, HaCaT, HEK 293, and skin fibroblasts. Compared with lipofectamine (positive control), LNPs and LNP-MNs permitted more homogeneous transfection (a higher proportion of fluorescent cells). LNPs and LNP-MNs can deliver both mRNA and FITC-dextran, while lipofectamine cannot deliver FITC-dextran into cells. In summary, DoE has allowed the optimization of the LNP-MN formulation for transdermal drug delivery and successfully delivered mRNA into mice at room temperature. This will be important for designing and manufacturing MN drug delivery systems on a large scale as well as for the short-term optimization of drug delivery systems tailored to different patient specificities.

## 4 Methods

### 4.1 Synthesis and Formulation Optimization of LNPs

LNPs were prepared by microfluidic devices (Suzhou Cchip Scientific Instrument Co., Ltd.). Lipids were DOTAP chloride (AVT, China), dipalmitoylphosphatidylcholine (DPPC; AVT, China), 1,2-distearoyl-*sn*-glycero-3-phosphoethanolamine-*N*-[methoxy-(polyethylene glycol)-2000] (DSPE-PEG(2000); AVT, China), and cholesterol (AVT, China). They were dissolved in ethanol in a molar ratio of 30:18:2:50 and rapidly combined with sodium citrate buffer (pH = 4) dissolving 52 μg/mL EGFP-mRNA (Dakewe) and sodium citrate buffer (pH = 6) dissolving 3 mg/mL FITC-dextran (Sigma-Aldrich), respectively. Nanosuspensions were obtained by passing them through the microfluidic chip at a flow rate of 1:3 between the organic and aqueous phases. The obtained mRNA-LNP and FITC-LNP nanosuspensions were dialyzed by PBS buffer in a Slide-A-Lyzer mini dialysis cup (10K MWCO, Thermo Scientific). Centrifuge tubes were filled with phosphate-buffered saline (PBS, Sigma-Aldrich) and spun on a rotary shaker for 18 h of dialysis (FITC-LNP especially). mRNA-LNP was dialyzed at 4 °C. The PBS buffer was replaced every 2 h, and finally, the dialyzed nanosuspension was collected.

A three-factor, two-level full factorial design was employed to examine the main effects and interactions of processing and formulation parameters using DesignExpert 13 (Table S1). The factors included, (A) the total flow rate, which influences the Reynolds number, and (B) the total lipid concentration, with response variables being the particle size and polydispersity index (PDI). Flow rates ranged from 1 to 8 mL/min, while lipid concentrations varied from 3 to 24 mg/mL. The model was further refined by incorporating six flow rate levels (1, 2, 3, 4, 6, and 8 mL/min) and four lipid concentration levels (3, 6, 12, and 24 mg/mL), resulting in 17 experimental conditions. The levels of each variable were denoted as −1, 0, and 1, with the center point tested five times (3 × 2^2^ + 5). Data from the surface response map were analyzed in DesignExpert 13 to validate the DoE model, with a *p* threshold of 0.0500 used to determine statistical significance.

The hydrodynamic diameter, PDI, and ζ potentials of LNPs were analyzed by dynamic light scattering (DLS; Malvern Zetasizer Nano). The morphology of LNPs was observed using transmission electron microscopy (TEM). Briefly, the LNP suspension was placed on a premade copper plate. A phosphotungstic acid solution with a concentration of 2.00% was prepared; an appropriate amount of dye solution was added dropwise at the same position of the copper screen after 30 min, and the excess dye solution was absorbed by filter paper after 1 min. All samples are processed in the same way before imaging under TEM (JEM-F200_TFEG, JEOL).

### 4.2 Quantification of the Drug Encapsulation Efficiency in LNPs

The Quant-iT Ribogreen RNA assay kit (Invitrogen, Thermo Fisher Scientific) was used to determine the RNA in LNPs. Samples were first diluted to 10 μg/mL in 1× TE buffer, and RiboGreen dye was added to measure the amount of unencapsulated mRNA in the LNPs. The samples were diluted in 1× TE buffer containing 0.5% (v/v) Triton X-100 (Sigma-Aldrich), and the total mRNA content was measured. The assay was carried out according to the manufacturer’s protocol. Fluorescence intensities were measured at an excitation wavelength of 480 nm and an emission wavelength of 520 nm. Standard curves are used to calculate the encapsulation dose based on the fluorescence intensity.

$$\mathrm{EE\%}=\frac{\mathrm{mass of encapsulated mRNA}}{\mathrm{total mass of mRNA used}}\times 100\%$$

To measure the quantity of FITC-dextran in FITC-dextran-LNP, various concentrations of FITC-dextran were dissolved in PBS buffer, and the calibration curve of the fluorescence intensity versus FITC-dextran concentration was plotted by a Microplate Reader. The FITC-dextran content in FITC-dextran-LNP before and after dialysis was obtained. The encapsulation rate was calculated as described above.

### 4.3 Fabrication and Optimization of mRNA-LNP-MNs and FITC-LNP-MNs

The PDMS MN negative molds were prepared as described in our previous work.^50,51^ The mold was subjected to plasma treatment (40 kHz, 0.1 mbar, 30 s) to enhance its hydrophilicity before usage. mRNA-LNP/FITC-LNP was mixed with PVP (Sigma-Aldrich, 40 000 Da) in a 2:1 ratio, which was poured into the MN mold. The air gap was removed by vacuum. Afterward, it was dried and placed in an Automatic Desiccator Cabinet.

A three-factor, two-level 2^2^ full factorial design was employed to examine the main effects and interactions of different processing and formulation parameters (Table S2). The factors were (A) the PVP concentration and (B) the oven drying temperature. Response factors (i.e., critical mass attributes) were Young’s modulus of MNs and the particle size of LNPs after their release from LNP-MNs. The levels of each variable are denoted as −1, 0, and 1. The experiments were conducted 13 times with a center point of 3 × 2^2^ + 1.

### 4.4 Cell Transfection with mRNA-LNP, FITC-Dextran-LNP, and LNP-MNs

HEK 293 cells, human embryonic stem cell (ESC)-derived MSCs, HaCaT cells, and skin fibroblast cells were provided by Ysbiotech (Hangzhou, China) and cultured at 37 °C in a humidified atmosphere of 5% CO_2_ in DMEM with 10% FBS (Thermo Fisher). Cells were placed into 6- or 12-well plates (30 cells/well), and 350 μL of the LNP nanosuspension was added. Similarly, LNP-MNs were dissolved in DMEM (1% FBS) and added to the cells. The negative control group consisted of 4 μg/mL mRNA or 3 mg/mL of FITC-dextran alone. The positive control consisted of 6 μL of lipofectamine 3000 (Thermo Fisher) combined with 4 μg/mL EGFP-mRNA or 3 mg/mL FITC-dextran. After incubation for 4–8 h, the medium was removed. Cells were washed using PBS three times and replenished with DMEM with 10% FBS for 24 h. The fluorescence intensity was observed under a Nikon fluorescence microscope with an excitation light source of 488 nm. For flow cytometry analysis (BD FACSVerse Cell Analyzer), cells were collected after 24 h of expression following transfection. The flow cytometry data were analyzed using FlowJo version 10.

### 4.5 Endocytosis Test

The steps of the endocytosis assay were the same as those of normal transfection, and a control experiment was set up during transfection. The first group of control HEK 293 cells was transfected according to the above steps, and the second group of control HEK 293 cells was transfected by being placed in a refrigerator at 4 °C for 40 min, rinsed with PBS, replaced with DMEM (10% FBS), and incubated at 37 °C for 10 min. This process was repeated twice, 2 h later. Finally, the fluorescence intensities of the two groups of cells were observed under a fluorescence microscope as shown in Figure S9.

### 4.6 FITC-Dextran-LNP Uptake and Endosomal Escape Test

MSCs were seeded in glass-bottom dishes and cultured at 37 °C with 5% CO_2_ to ∼70% confluence. FITC-dextran-LNPs were then added and incubated for 1, 3, or 12 h. Thirty minutes before the end of each incubation, LysoTracker Deep Red (Thermo Fisher, L12492; 50 nM) and Hoechst 33342 (Thermo Fisher, H3570; 1 μg/mL) were added simultaneously. After staining, cells were washed twice with prewarmed PBS and imaged live on a Leica TCS SP8 confocal microscope at 37 °C. Z-Stack images were acquired with excitation wavelengths of 488, 650, and 405 nm (scale bar of 10 μm) (Figure S10).

### 4.7 Skin Penetration Test

Pig ear skin was sourced from a local market, rinsed in deionized water, shaved, and cleared of subcutaneous fat. The MN patch was pressed into the dermal surface for 5 min and then removed. Skin samples were embedded in OCT, frozen at −80 °C, and cryosectioned at 10 μm thickness to capture the microneedle penetration depth. Sections were air-dried, fixed in cold acetone for 10 min, rehydrated through graded ethanol, and stained with hematoxylin (5 min) followed by eosin (2 min). After dehydration and clearing, slides were mounted and imaged to visualize microchannels created by MN insertion via H&E contrast.

### 4.8 Microneedle SEM Characterization

The microneedle arrays were affixed to an SEM stub using conductive adhesive and sputter-coated with a 5–10 nm layer of gold to minimize sample charging. Under vacuum (10^–5^–10^–6^ Torr), samples were imaged in secondary electron mode at an acceleration voltage of 5–10 kV and a working distance of 5–10 mm, capturing both the overall array arrangement and needle tip details (FEI Quanta 250 e-SEM). Images were exported as high-resolution TIFFs and annotated with scale bars as needed.

### 4.9 Cell Transfection under a 3D Environment Using LNP-MNs

MSC suspension of 1 × 10^6^ cells/mL were mixed with VitroGel MSC in a 1:2 ratio. The mixture was gently pipetted up and down for 5–10 times and transferred to 24-well plates (300 μL per well). Ten to fifteen minutes later, an additional 300 μL of cell medium was added to cover the hydrogel. After 10 days of culture, the culture medium was replaced with the serum-free culture medium. mRNA-LNP-MN was applied for 4 h. Afterward, a complete medium was added and cells were cultured for 24 h before fluorescence imaging and flow cytometry analysis.

### 4.10 Assessment of mRNA Expression in Mice

. The animal experiments involving MNtransdermal administration of MNs were approved by the Animal Research Ethics Sub-committee of the City University of Hong Kong (reference no. AN-STA-00000706).

Consistent with the above EGFP mRNA-LNP-MN preparation process, Fluc mRNA-LNP-MNPs were prepared with Fluc mRNA at a dose of 4.0 μg of mRNA per microneedle patch. Five or six patches were applied to the dorsal surface of each mouse and rapidly lanced into the dorsal epidermis and pressed for 1 min to dissolve, with mRNA applied at a dose of 20–24 μg per mouse, the same as in the EGFP expression study described above (*n* = 3). The MNPs were stored in dry collection boxes at room temperature. Photographs were taken 24 h after MNP administration with the IVIS instrument.

### 4.11 Statistical Analysis

All data were analyzed with GraphPad Prism version 9.0 (GraphPad Software Inc.) and expressed as the mean ± standard error of the mean. The difference between groups was tested using either one-way or two-way analysis of variance (ANOVA). Specific statistical analysis methods are described in the figure legends, where results are presented. Values were considered statistically significant for *p* < 0.05.

## Supplementary Material



## Notes

Schematic illustration of LNPs (Figure S1), MSC transfection by FITC-dextran-LNPs (Figure S2), fluorescence images of skin fibroblasts and HEK 293 cells transfected with FITC-dextran-LNPs (Figure S3), schematic illustration of LNP-MN patch fabrication (Figure S4), compression test force–displacement curves for LNP-MNs with various PVP concentrations at 20, 40, and 60 °C (Figure S5), schematic of the *in vitro* 2D MSC transfection workflow using EGFP-mRNA-LNP-MNs (Figure S6), MSC transfection by FITC-dextran-LNP-MNs (Figure S7), fluorescence images of the transfection of MN at different PVP concentrations in skin fibroblasts (Figure S8), comparison of fluorescence images of FITC-dextran-LNP endocytosis inhibited by low temperatures in HEK293 cells (Figure S9), time-dependent confocal imaging of FITC-dextran-LNP uptake and endosomal escape in MSCs (Figure S10), formulation parameters of lipid nanoparticles (Table S1), and formulation parameters of the LNP-MN patch (Table S2) (PDF)

## References

[1] Tenchov R., Bird R., Curtze A. E., Zhou Q. (2021). Lipid Nanoparticles
horizontal line From Liposomes to mRNA Vaccine Delivery, a Landscape
of Research Diversity and Advancement. ACS Nano.

[2] Hou X., Zaks T., Langer R., Dong Y. (2021). Lipid nanoparticles
for mRNA delivery. Nature Reviews Materials.

[3] Liu Y., Huang Y., He G., Guo C., Dong J., Wu L. (2024). Development of mRNA
Lipid Nanoparticles: Targeting and Therapeutic
Aspects. Int. J. Mol. Sci..

[4] Ruan S., Zhang Y., Feng N. (2021). Microneedle-mediated
transdermal
nanodelivery systems: a review. Biomater Sci..

[5] Tavares
Luiz M., Santos Rosa Viegas J., Palma Abriata J., Viegas F., Testa Moura de Carvalho Vicentini F., Lopes Badra Bentley M. V., Chorilli M., Maldonado
Marchetti J., Tapia-Blacido D. R. (2021). Design of experiments (DoE) to develop
and to optimize nanoparticles as drug delivery systems. Eur. J. Pharm. Biopharm.

[6] Gurba-Bryskiewicz L., Maruszak W., Smuga D. A., Dubiel K., Wieczorek M. (2023). Quality by
Design (QbD) and Design of Experiments (DOE) as a Strategy for Tuning
Lipid Nanoparticle Formulations for RNA Delivery. Biomedicines.

[7] Zhang T., Yin H., Li Y., Yang H., Ge K., Zhang J., Yuan Q., Dai X., Naeem A., Weng Y., Huang Y., Liang X. J. (2024). Optimized lipid nanoparticles (LNPs)
for organ-selective nucleic acids delivery in vivo. iScience.

[8] Anselmo A. C., Gokarn Y., Mitragotri S. (2019). Non-invasive
delivery strategies
for biologics. Nat. Rev. Drug Discov.

[9] Vander
Straeten A., Sarmadi M., Daristotle J. L., Kanelli M., Tostanoski L. H., Collins J., Pardeshi A., Han J., Varshney D., Eshaghi B., Garcia J., Forster T. A., Li G., Menon N., Pyon S. L., Zhang L., Jacob-Dolan C., Powers O. C., Hall K., Alsaiari S. K., Wolf M., Tibbitt M. W., Farra R., Barouch D. H., Langer R., Jaklenec A. (2024). A microneedle vaccine printer for thermostable COVID-19
mRNA vaccines. Nat. Biotechnol..

[10] Qu F., Sun Y., Bi D., Peng S., Li M., Liu H., Zhang L., Tao J., Liu Y., Zhu J. (2023). Regulating
Size and Charge of Liposomes in Microneedles to Enhance Intracellular
Drug Delivery Efficiency in Skin for Psoriasis Therapy. Adv. Healthcare Mater..

[11] Nguyen H. X. (2025). Beyond
the Needle: Innovative Microneedle-Based Transdermal Vaccination. Medicines.

[12] Anbazhagan G., Suseela S. B., Sankararajan R. (2023). Design, analysis
and fabrication
of solid polymer microneedle patch using CO(2) laser and polymer molding. Drug Deliv Transl Res..

[13] Jiang X., Zhao H., Li W. (2022). Microneedle-Mediated
Transdermal
Delivery of Drug-Carrying Nanoparticles. Front
Bioeng Biotechnol.

[14] Ferreira N. N., Miranda R. R., Moreno N. S., Pincela Lins P. M., Leite C. M., Leite A. E. T., Machado T. R., Cataldi T. R., Labate C. A., Reis R. M., Zucolotto V. (2023). Using design
of experiments (DoE) to optimize performance and stability of biomimetic
cell membrane-coated nanostructures for cancer therapy. Front. Bioeng. Biotechnol..

[15] Abdullah A. C., Ahmadinejad E., Tasoglu S. (2024). Optimizing Solid Microneedle
Design:
A Comprehensive ML-Augmented DOE Approach. ACS
Meas Sci. Au.

[16] Safford H. C., Swingle K. L., Geisler H. C., Hamilton A. G., Thatte A. S., Ghalsasi A. A., Billingsley M. M., Alameh M. G., Weissman D., Mitchell M. J. (2023). Orthogonal Design
of Experiments for Engineering of
Lipid Nanoparticles for mRNA Delivery to the Placenta. Small.

[17] Held J., Gaspar J., Ruther P., Hagner M., Cismak A., Heilmann A., Paul O. (2010). Design of
experiment characterization
of microneedle fabrication processes based on dry silicon etching. J. Micromech. Microeng..

[18] Matsuura-Sawada Y., Maeki M., Nishioka T., Niwa A., Yamauchi J., Mizoguchi M., Wada K., Tokeshi M. (2022). Microfluidic
Device-Enabled
Mass Production of Lipid-Based Nanoparticles for Applications in Nanomedicine
and Cosmetics. ACS Applied Nano Materials.

[19] Xu L., Wang X., Liu Y., Yang G., Falconer R. J., Zhao C.-X. (2022). Lipid nanoparticles
for drug delivery. Adv. NanoBiomed Res..

[20] Binici B., Rattray Z., Zinger A., Perrie Y. (2025). Exploring the impact
of commonly used ionizable and pegylated lipids on mRNA-LNPs: A combined
in vitro and preclinical perspective. J. Controlled
Release.

[21] Chen H., Ren X., Xu S., Zhang D., Han T. (2022). Optimization of Lipid
Nanoformulations for Effective mRNA Delivery. Int. J. Nanomedicine.

[22] Sun D., Lu Z. R. (2023). Structure and Function of Cationic and Ionizable Lipids for Nucleic
Acid Delivery. Pharm. Res..

[23] Wang J., Ding Y., Chong K., Cui M., Cao Z., Tang C., Tian Z., Hu Y., Zhao Y., Jiang S. (2024). Recent Advances in Lipid Nanoparticles
and Their Safety Concerns
for mRNA Delivery. Vaccines.

[24] Shepherd S. J., Han X., Mukalel A. J., El-Mayta R., Thatte A. S., Wu J., Padilla M. S., Alameh M.-G., Srikumar N., Lee D. (2023). Throughput-scalable
manufacturing of SARS-CoV-2 mRNA lipid nanoparticle
vaccines. Proc. Natl. Acad. Sci. U. S. A..

[25] Nag K., Sarker M. E. H., Kumar S., Khan H., Chakraborty S., Islam M. J., Baray J. C., Khan M. R., Mahmud A., Barman U. (2022). DoE-derived
continuous and robust process for manufacturing
of pharmaceutical-grade wide-range LNPs for RNA-vaccine/drug delivery. Sci. Rep..

[26] Vogelaar A., Marcotte S., Cheng J., Oluoch B., Zaro J. (2023). Use of Microfluidics
to Prepare Lipid-Based Nanocarriers. Pharmaceutics.

[27] Weng J., Shao Z., Chan H. W., Li S. P. Y., Lam J. K. W., Tsang C. K., Chow S. (2022). Mediating
bio-fate of polymeric cholecalciferol
nanoparticles through rational size control. Biomater. Adv..

[28] Young R. E., Nelson K. M., Hofbauer S. I., Vijayakumar T., Alameh M. G., Weissman D., Papachristou C., Gleghorn J. P., Riley R. S. (2024). Systematic development of ionizable
lipid nanoparticles for placental mRNA delivery using a design of
experiments approach. Bioact Mater..

[29] Ripoll M., Martin E., Enot M., Robbe O., Rapisarda C., Nicolai M. C., Deliot A., Tabeling P., Authelin J. R., Nakach M., Wils P. (2022). Optimal self-assembly
of lipid nanoparticles
(LNP) in a ring micromixer. Sci. Rep..

[30] Terada T., Kulkarni J. A., Huynh A., Chen S., van der
Meel R., Tam Y. Y. C., Cullis P. R. (2021). Characterization of Lipid Nanoparticles
Containing Ionizable Cationic Lipids Using Design-of-Experiments Approach. Langmuir.

[31] Maeki M., Fujishima Y., Sato Y., Yasui T., Kaji N., Ishida A., Tani H., Baba Y., Harashima H., Tokeshi M. (2017). Understanding the formation mechanism of lipid nanoparticles
in microfluidic devices with chaotic micromixers. PLoS One.

[32] Jiang N., Tian X., Wang Q., Hao J., Jiang J., Wang H. (2024). Regulation Mechanisms and Maintenance
Strategies of Stemness in Mesenchymal
Stem Cells. Stem Cell Rev. Rep.

[33] Seo M. D., Kang T. J., Lee C. H., Lee A. Y., Noh M. (2012). HaCaT Keratinocytes
and Primary Epidermal Keratinocytes Have Different Transcriptional
Profiles of Cornified Envelope-Associated Genes to T Helper Cell Cytokines. Biomol Ther (Seoul).

[34] Hazrati R., Davaran S., Keyhanvar P., Soltani S., Alizadeh E. (2024). A Systematic
Review of Stem Cell Differentiation into Keratinocytes for Regenerative
Applications. Stem Cell Rev. Rep.

[35] Paunovska K., Da Silva Sanchez A., Foster M. T., Loughrey D., Blanchard E. L., Islam F. Z., Gan Z., Mantalaris A., Santangelo P. J., Dahlman J. (2020). Increased PIP3 activity blocks nanoparticle
mRNA delivery. Sci. Adv..

[36] Herrera M., Kim J., Eygeris Y., Jozic A., Sahay G. (2021). Illuminating endosomal
escape of polymorphic lipid nanoparticles that boost mRNA delivery. Biomater Sci..

[37] Takikawa M., Fujisawa M., Yoshino K., Takeoka S. (2020). Intracellular Distribution
of Lipids and Encapsulated Model Drugs from Cationic Liposomes with
Different Uptake Pathways. Int. J. Nanomedicine.

[38] Zhang L., More K. R., Ojha A., Jackson C. B., Quinlan B. D., Li H., He W., Farzan M., Pardi N., Choe H. (2023). Effect of
mRNA-LNP components of two globally-marketed COVID-19 vaccines on
efficacy and stability. npj Vaccines.

[39] Koh K. J., Liu Y., Lim S. H., Loh X. J., Kang L., Lim C. Y., Phua K. (2018). Formulation,
characterization and evaluation of mRNA-loaded dissolvable
polymeric microneedles (RNApatch). Sci. Rep..

[40] Luo Y., Hong Y., Shen L., Wu F., Lin X. (2021). Multifunctional
Role of Polyvinylpyrrolidone in Pharmaceutical Formulations. AAPS PharmSciTech.

[41] Nair K., Whiteside B., Grant C., Patel R., Tuinea-Bobe C., Norris K., Paradkar A. (2015). Investigation of plasma
treatment
on micro-injection moulded microneedle for drug delivery. Pharmaceutics.

[42] Held J., Gaspar J., Ruther P., Hagner M., Cismak A., Heilmann A., Paul O. J. (2010). Design of experiment
characterization
of microneedle fabrication processes based on dry silicon etching. J. Micromech. Microeng..

[43] Oh N. G., Hwang S. Y., Na Y. H. (2022). Fabrication of a
PVA-Based Hydrogel
Microneedle Patch. ACS Omega.

[44] Chang H., Chew S. W., Zheng M., Lio D. C. S., Wiraja C., Mei Y., Ning X., Cui M., Than A., Shi P. (2021). Cryomicroneedles for
transdermal cell delivery.. Nat. Biomed. Eng..

[45] Jacobi U., Kaiser M., Toll R., Mangelsdorf S., Audring H., Otberg N., Sterry W., Lademann J. (2007). Porcine ear
skin: an in vitro model for human skin. Skin
Res. Technol..

[46] Powell K. (2017). Adding depth
to cell culture. Science.

[47] Wang Y., Li Z., Ouyang J., Karniadakis G. E. (2020). Controlled release of entrapped nanoparticles
from thermoresponsive hydrogels with tunable network characteristics. Soft Matter.

[48] Su C., Lin D., Huang X., Feng J., Jin A., Wang F., Lv Q., Lei L., Pan W. (2024). Developing hydrogels for gene therapy
and tissue engineering. J. Nanobiotechnol..

[49] Mdanda S., Ubanako P., Kondiah P. P., Kumar P., Choonara Y. E. (2021). Recent
advances in microneedle platforms for transdermal drug delivery technologies. Polymers.

[50] Chang H., Wen X., Li Z., Ling Z., Zheng Y., Xu C. (2023). Co-delivery
of dendritic cell vaccine and anti-PD-1 antibody with cryomicroneedles
for combinational immunotherapy. Bioeng. Transl.
Med..

[51] Zheng M., Zhang Y., Hu T., Xu C. (2023). A skin patch integrating
swellable microneedles and electrochemical test strips for glucose
and alcohol measurement in skin interstitial fluid. Bioeng. Transl. Med..

