# Supplementary material for: Optimization of a Dissolvable Lipid Nanoparticle Microneedle Formulation for mRNA Delivery Using Design of Experiments
Source: ACS Appl Mater Interfaces. 2025 Jul 11;17(29):41597–609. doi: 10.1021/acsami.5c05528 (PMC12713615; doi:10.1021/acsami.5c05528)
Supplement: Supplementary file 1 [file am5c05528_si_001.pdf]

Supporting Information

Optimization of Dissolvable Lipid Nanoparticle Microneedle Formulation for mRNA Delivery Using Design of Experiments

Yayi Zhao[1], Qizheng Zhang[1,4], Tianli Hu[1], Chaiyaporn Kuwentrai[2], Ye Zhang[4], Jian-Dong Huang[2], Yi Kuang[3], Chenjie Xu[1,5*]

1. Department of Biomedical Engineering, College of Biomedicine, City University of Hong Kong, Tat Chee Avenue.Kowloon, Hong Kong SAR, China

2. School of Biomedical Sciences, Li Ka Shing Faculty of Medicine, The University of Hong Kong, Pokfulam, Hong Kong SAR, China

3. Department of Chemical and Biological Engineering, The Hong Kong University of Science and Technology, Clear Water Bay, Kowloon, Hong Kong SAR, China

4. Active Soft Matter Group, Songshan Lake Materials Laboratory, Dongguan, Guangdong 523808, China

5. Institute of Digital Medicine, City University of Hong Kong, Tat Chee Avenue, Kowloon, Hong Kong SAR, China

*Email: chenjie.xu@cityu.edu.hk

Table S1. The formulation parameters of lipid nanoparticles.

	Factor 1	Factor 2	Response 1	Response 2
Run	Total lipid con. (mg/ml)	TFR (ml/min)	Particle Size (nm)	PDI
1	12	8	80	0.215
2	12	6	128.8	0.175
3	12	4	143.3	0.164
4	12	2	192.8	0.177
5	24	8	125	0.343
6	24	6	135.2	0.22
7	24	4	145	0.188
8	24	2	165	0.073
9	6	6	110	0.234
10	6	4	135	0.219
11	6	2	191.3	0.133
12	6	3	159.7	0.235
13	6	1	187.3	0.148
14	3	4	93.6	0.106
15	3	3	100.5	0.11
16	3	2	102.4	0.087
17	3	1	190.4	0.129

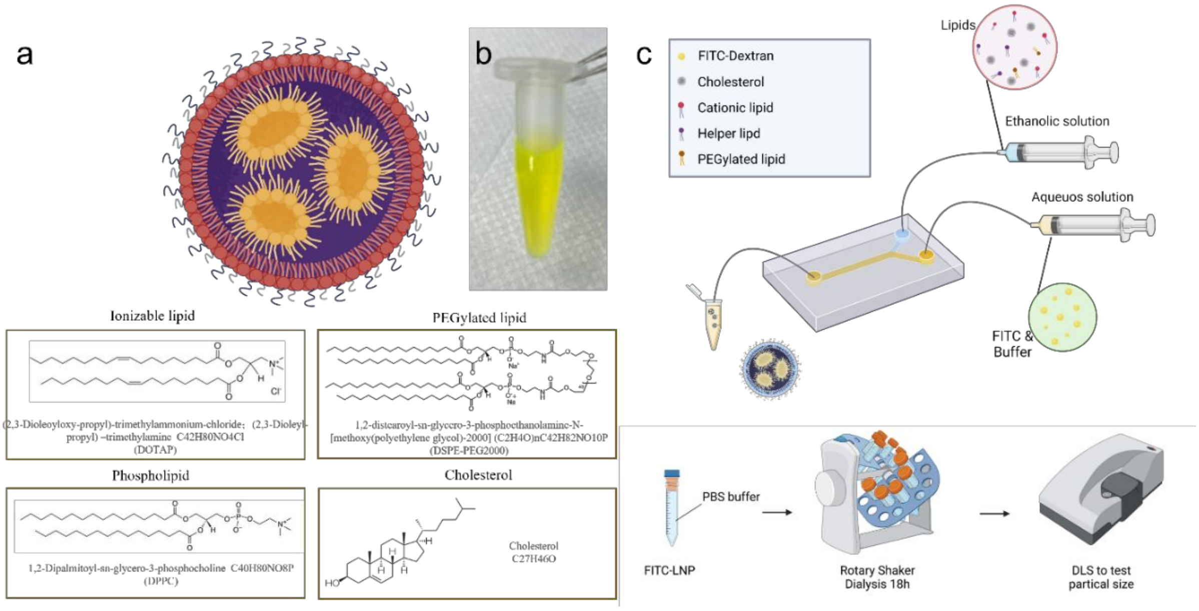

Figure S1. LNP fabrication: a) LNP components; b) Photo of FITC-dextran-LNP solution; c) Microfluidic synthesis of FITC-dextran-LNPs. The cartoon was created in BioRender. Xu, C.(2024) https://BioRender.com/d93u393.

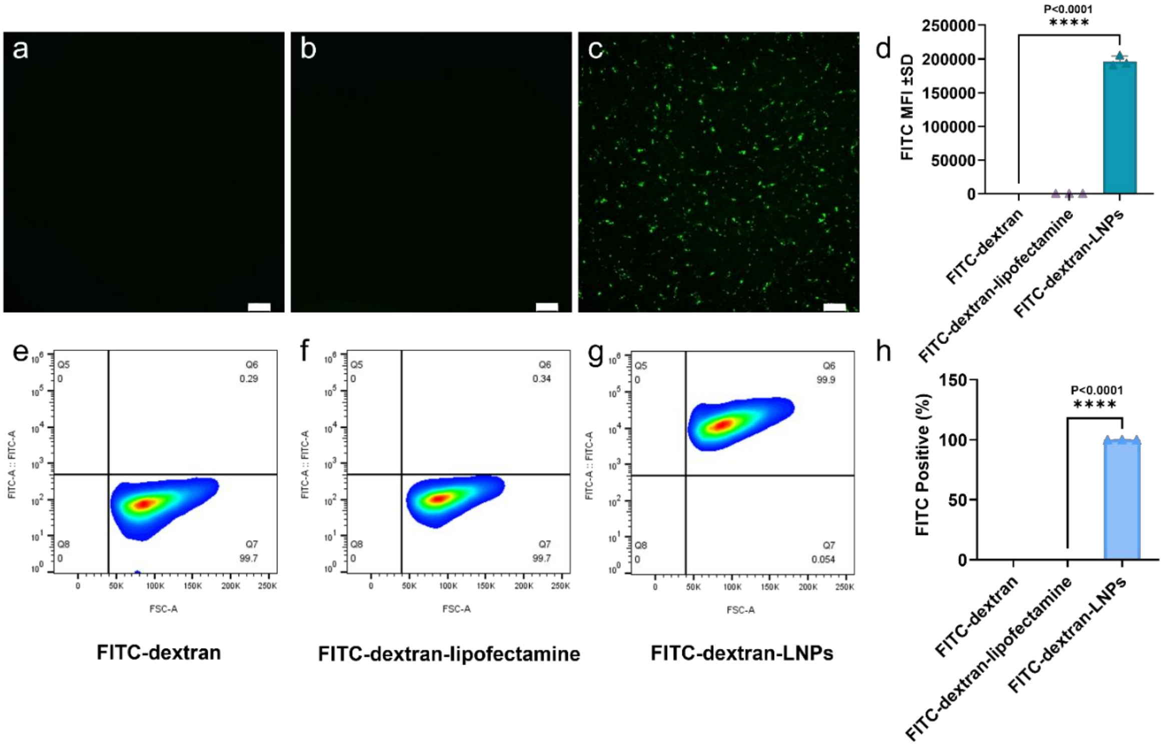

Figure S2. MSC transfection by FITC-dextran-LNPs: Fluorescence imaging of MSCs transfected with a) FITC-dextran alone, b) FITC-dextran-lipofectiome, c) FITC-dextran-LNPs, scale bar: 250 μm; d) quantification of average fluorescence per fluorescent cell in the fluorescence image; flow cytometry analysis of MSCs transfected with e) FITC-dextran alone, f) FITC-dextran-lipofectiome, g) FITC-dextran-LNPs; h) quantification of fluorescence intensity of cells in flow cytometry. Values are expressed as mean ± SD. Error bars indicate SD values from three independent experiments. Statistical differences are expressed as **$p < 0.005$, ***$p < 0.001$.

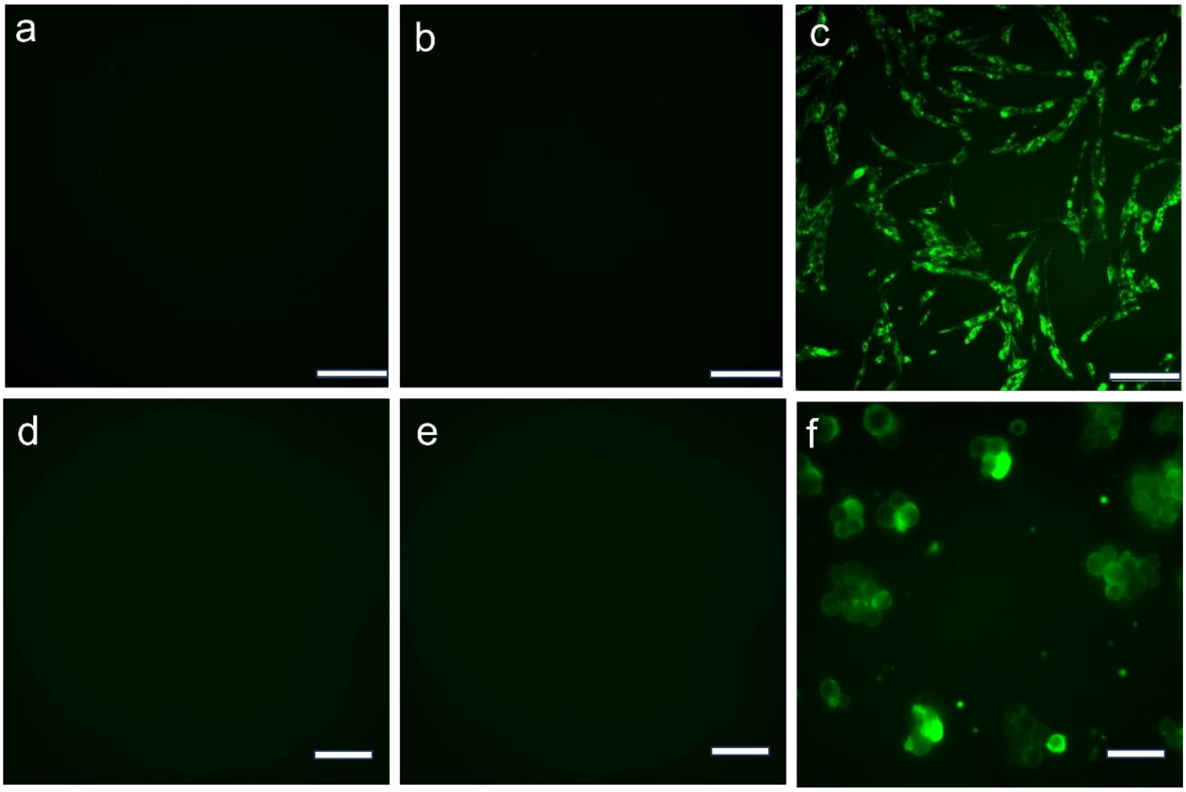

Figure S3. Skin fibroblasts transfected with a) FITC-dextran alone, b) FITC-dextran-lipofectamine, c) FITC-dextran-LNP, Scale bar: 250 µm; HEK 293 cells transfected with d) FITC-dextran alone, e) FITC-dextran-lipofectamine, f) FITC-dextran-LNPs, scale bar: 50 µm.

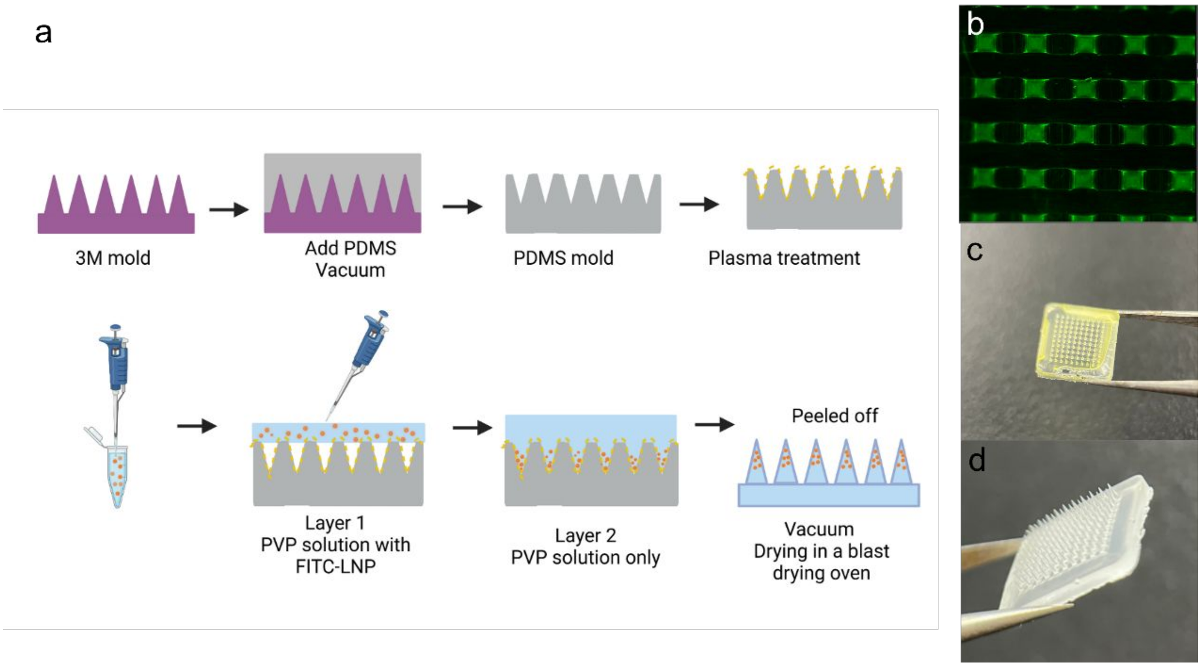

Figure S4. LNP-MN patch fabrication: a) Illustration of the fabrication process of LNP-MNs; b) Fluorescence image and c) photo of FITC-dextran-LNP-MNs; d) Photo of EGFP-mRNA-LNP-MN patch.

Table S2. The formulation parameters of LNP-MN patch.

	Factor 1	Factor 2	Response 1	Response 2
Run	PVP concentration (%)	Temperature (℃)	Size (nm)	Young's modulus (mPa)
1	10	20	356.07	299.7118156
2	15	20	285.3	258.6320651
3	20	20	253	493.926725
4	25	20	98.18	433.824543
5	30	20	116.7	353.9478802
6	10	40	445.1	786.7309494
7	15	40	286.7	771.7863928
8	20	40	223.8	316.7541766
9	25	40	181.9	302.6499261
10	30	40	324.9	964.6856158
11	10	60	422.1	396.4696189
12	15	60	509.2	748.5008696
13	20	60	98.54	440.0041477
14	25	60	130.1	920.6298763
15	30	60	47.37	633.0434783

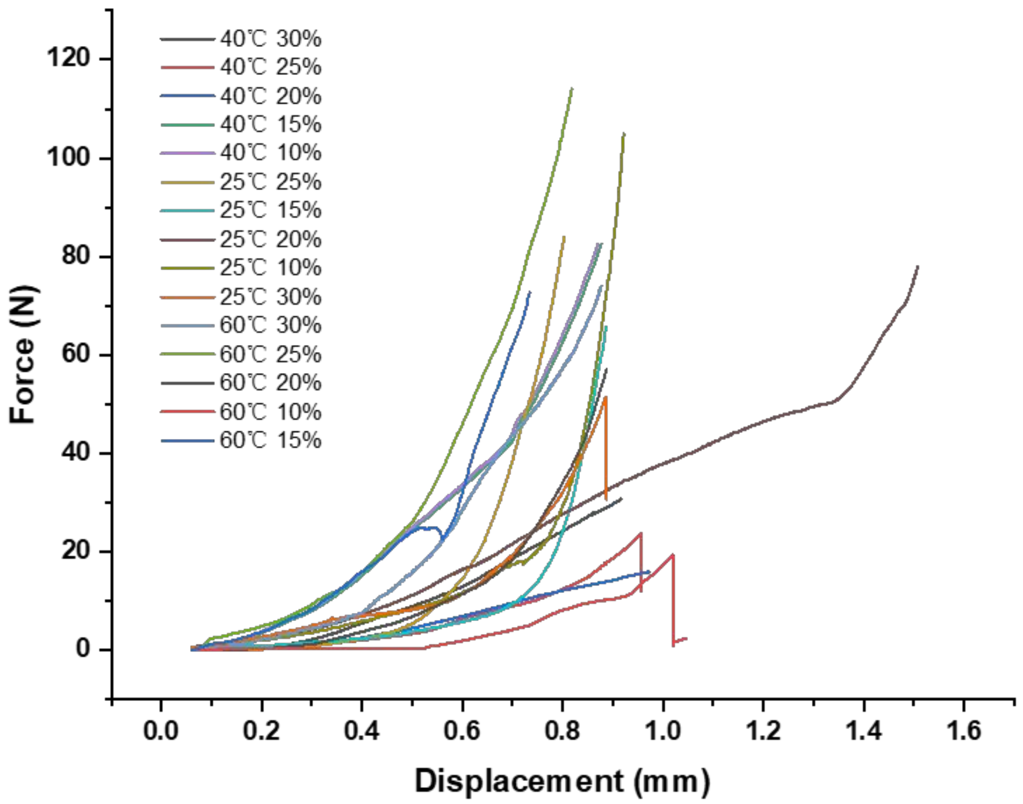

Figure S5. The force-displacement curve of compression test of LNP-MNs that were made of different PVP concentrations under three drying temperatures (20℃, 40℃, 60℃)

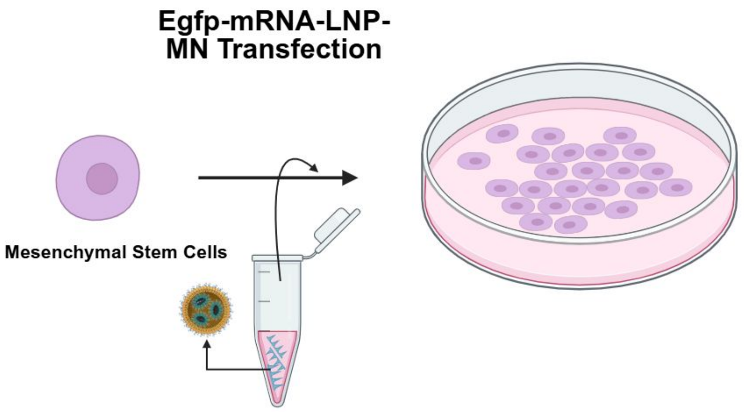

Figure S6. Schematic of *in vitro* 2D MSC transfection workflow using EGFP-mRNA–LNP-MN. The cartoon was created in BioRender. Xu, C.(2024) https://BioRender.com/d93u393.

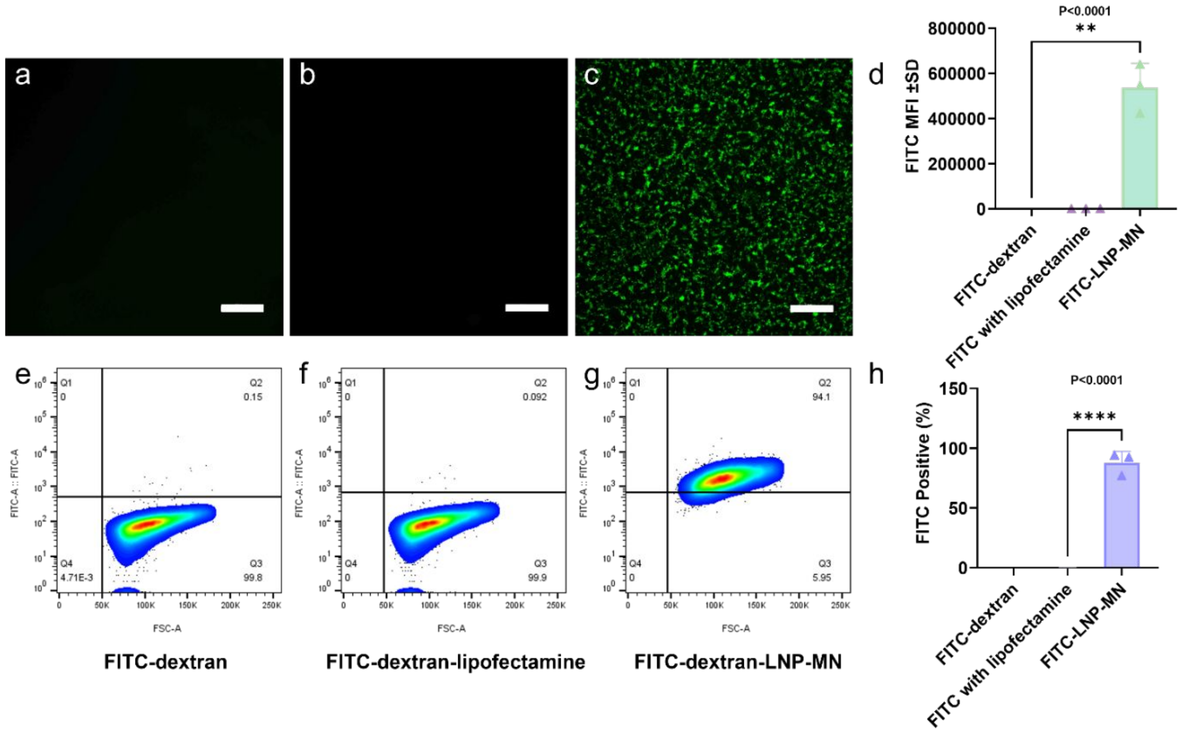

Figure S7. MSC transfection by FITC-dextran-LNP-MNs: Fluorescence imaging of MSCs transfected with a) FITC-dextran alone, b) FITC-dextran-lipofectamine, c) FITC-dextran-LNP-MNs, scale bar: 500 µm; d) quantification of average fluorescence per fluorescent cell in the fluorescence image; flow cytometry analysis of MSCs transfected with e) FITC-dextran alone, f) FITC-dextran-lipofectamine, g) FITC-dextran-LNP-MNs; h) quantification of fluorescence intensity of cells in flow cytometry. Values are expressed as mean ± SD. Error bars indicate SD values from three independent experiments. Statistical differences are expressed as **$p < 0.005$, ***$p < 0.001$.

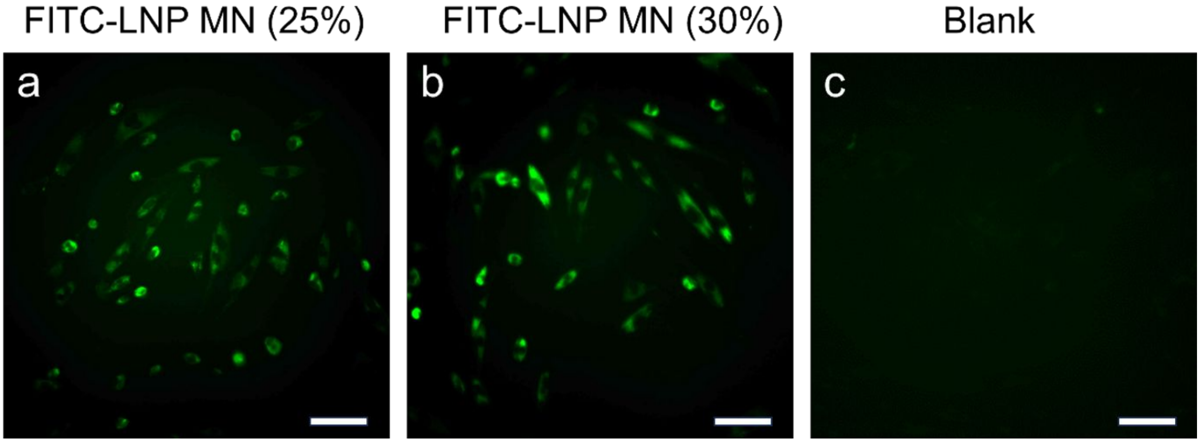

Figure S8. Fluorescence images of skin fibroblasts transfected with a) FITC-dextran-LNP-MNs fabricated with 25% of PVP after drying at 60°C, b) FITC-dextran-LNP-MNs fabricated with 30% of PVP after drying at 60°C, and c) FITC-dextran alone, scale bar: 50 μm.

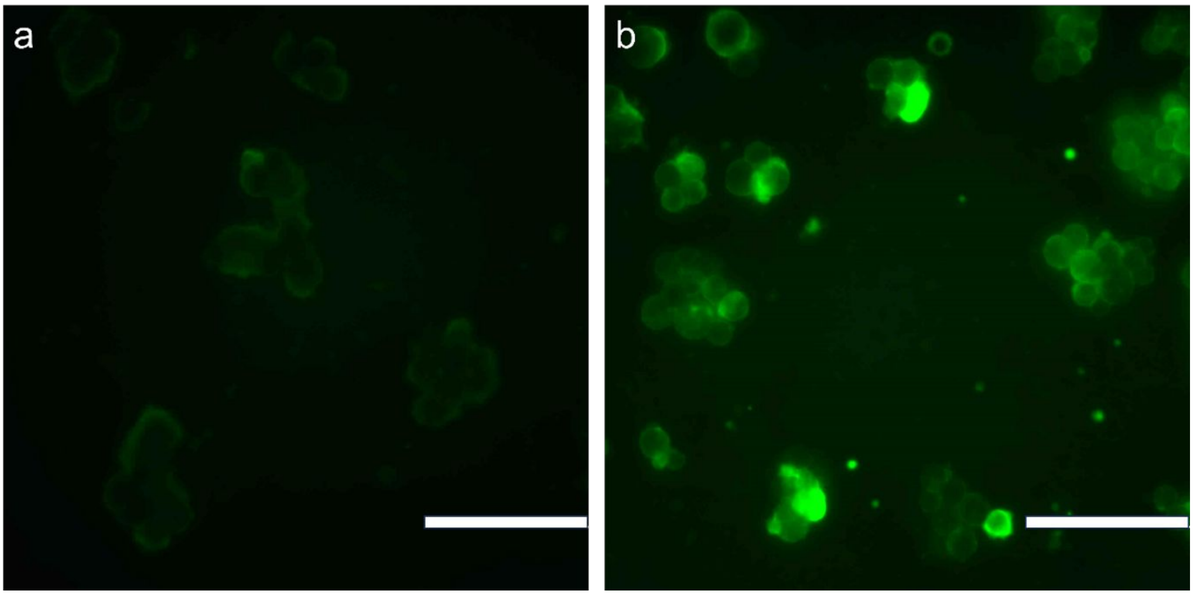

Figure S9. Fluorescence images of HEK293 transfected with a) 50% volume of FITC-dextran-LNP with DMEM (1% FBS) for 2 hours at 4 °C. b) 50% volume of FITC-dextran-LNP with DMEM (1% FBS) for 2 hours at 37 °C, scale bar: 100 μm.

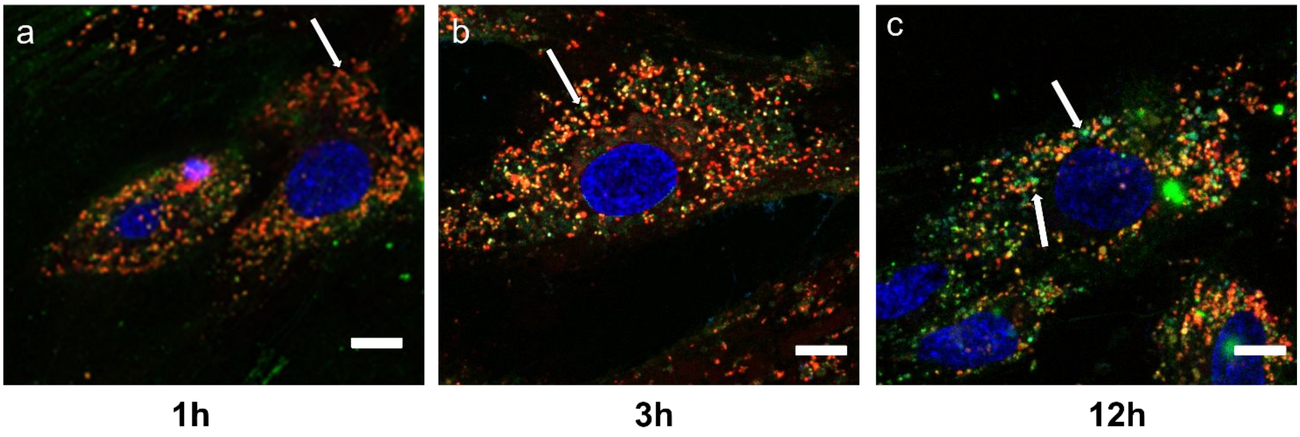

Figure S10. Time-dependent confocal imaging of FITC–Dextran–LNP uptake and endosomal escape in MSCs a) Fluorescence images of FITC-Dextran-LNP and MSC incubated for 1 h under the same treatment; b) Fluorescence images of FITC-Dextran-LNP and MSC incubated for 3 hours under the same treatment; c) Fluorescence images of FITC-Dextran-LNP and MSC incubated for 12 hours under the same treatment. scale bar: 10 µm.
